# Activity of Thioallyl Compounds From Garlic Against *Giardia duodenalis* Trophozoites and in Experimental Giardiasis

**DOI:** 10.3389/fcimb.2018.00353

**Published:** 2018-10-15

**Authors:** Raúl Argüello-García, Mariana de la Vega-Arnaud, Iraís J. Loredo-Rodríguez, Adriana M. Mejía-Corona, Elizabeth Melgarejo-Trejo, Eulogia A. Espinoza-Contreras, Rocío Fonseca-Liñán, Arturo González-Robles, Nury Pérez-Hernández, M. Guadalupe Ortega-Pierres

**Affiliations:** ^1^Departamento de Genética y Biología Molecular, Centro de Investigación y de Estudios Avanzados del Instituto Politécnico Nacional, Mexico City, Mexico; ^2^Departamento de Infectómica y Patogénesis Experimental, Centro de Investigación y de Estudios Avanzados del Instituto Politécnico Nacional, Mexico City, Mexico; ^3^Escuela Nacional de Medicina y Homeopatía, Instituto Politécnico Nacional, Mexico City, Mexico

**Keywords:** *Giardia duodenalis*, trophozoite damage, garlic, thioallyl compounds, allicin, giardiasis

## Abstract

Fresh aqueous extracts (AGEs) and several thioallyl compounds (TACs) from garlic have an important antimicrobial activity that likely involves their interaction with exposed thiol groups at single aminoacids or target proteins. Since these groups are present in *Giardia duodenalis* trophozoites, in this work we evaluated the anti-giardial activity of AGE and several garlic's TACs. *In vitro* susceptibility assays showed that AGE affected trophozoite viability initially by a mechanism impairing cell integrity and oxidoreductase activities while diesterase activities were abrogated at higher AGE concentrations. The giardicidal activities of seven TACs were related to the molecular descriptor HOMO (Highest Occupied Molecular Orbital) energy and with their capacity to modify the –SH groups exposed in giardial proteins. Interestingly, the activity of several cysteine proteases in trophozoite lysates was inhibited by representative TACs as well as the cytopathic effect of the virulence factor giardipain-1. Of these, allicin showed the highest anti-giardial activity, the lower HOMO value, the highest thiol-modifying activity and the greatest inhibition of cysteine proteases. Allicin had a cytolytic mechanism in trophozoites with subsequent impairment of diesterase and oxidoreductase activities in a similar way to AGE. In addition, by electron microscopy a marked destruction of plasma membrane and endomembranes was observed in allicin-treated trophozoites while cytoskeletal elements were not affected. In further flow cytometry analyses pro-apoptotic effects of allicin concomitant to partial cell cycle arrest at G2 phase with the absence of oxidative stress were observed. In experimental infections of gerbils, the intragastric administration of AGE or allicin decreased parasite numbers and eliminated trophozoites in experimentally infected animals, respectively. These data suggest a potential use of TACs from garlic against *G. duodenalis* and in the treatment of giardiasis along with their additional benefits in the host's health.

## Introduction

Giardiasis is the intestinal parasitosis of highest incidence in developed countries with a global distribution of 280 million cases per year. Nearly 200 million people with symptomatic infections have been reported in Asia, Africa and Latin America where some 500,000 new cases per year are reported (Adam, 2001; Lane and Lloyd, 2002; WHO, 2006). Infection initiates when a vertebrate host consumes water or food containing infective cysts of *Giardia duodenalis* (syn. *Giardia lamblia, Giardia intestinalis*) and in the acidic gastric environment excystation is induced releasing excyzoites that divide to form trophozoites. These latter adhere to duodenum and jejunum epithelia triggering the pathogenic onset associated to giardiasis. At jejunum-ileum lumen trophozoites are exposed to high bile and low cholesterol concentrations at a slightly alkaline pH conditions that induce encystation. Cysts are shed in feces where they mature and remain viable for long periods of time until these are ingested by a new host. This infection may be symptomatic with acute signals characterized by diarrhea and abdominal complaints that may lead to malabsorption syndrome eventually associated to chronic outcomes. However, asymptomatic carriers have been found as a major group in hyperendemic areas (Redlinger et al., 2002).

The main strategy for the control of infections caused by *G. duodenalis*, comprises the use of a variety of antimicrobial drugs including acridines, (quinacrine), aminoglycosides (paromomycin) and most commonly drugs uses are 5-nitroheterocyclic compounds [metronidazole (MTZ), furazolidone, and nitazoxanide] and benzimidazoles [albendazole (ABZ) and mebendazole]. Most of these drugs have mild-to-severe secondary effects (revised by Gardner and Hill, 2001). Further, the use of sublethal concentrations of antigiardial drugs *in vitro* or suboptimal therapy regimes, which include single-dose regimes of benzimidazoles given in communitarian programs to deworm children in endemic localities, may derive into selection of drug resistant parasites and increased incidences of giardiasis (Northrop-Clewes et al., 2001; Argüello-García et al., 2004). Thereby a better knowledge of therapeutic targets in *Giardia* is needed to develop more effective drugs for the treatment of giardiasis. In this context, compounds derived from natural products such as garlic (*Allium sativum* L.), may be considered as promising and alternative perspective for treatment of giardiasis (Anthony et al., 2005).

Garlic, a perennial crop common in the human diet, has been used since ancient times in folk medicine of different cultures as a panacea. Likewise aqueous and oil extracts of garlic bulbs provide benefits in the prevention and treatment of gastrointestinal disorders including carcinogenesis and some infections (Ankri and Mirelman, 1999; Anthony et al., 2005; Bhagyalakshmi et al., 2007), however the basis of its effectiveness have not yet been completely unraveled. In this context, there are reports on the anti-tumoral activities of a number of organosulfur compounds contained or derived from garlic. This is most likely due to the modulatory activity of these compounds on carcinogen metabolizing enzymes combined with the pro-apoptotic, antioxidant and radical scavenging properties (Milner, 2001; Yang et al., 2001; Argüello-García et al., 2010; De Gianni and Fimognari, 2015). Also, garlic compounds were reported to have a direct activity against bacterial, protozoan, fungal and viral pathogens (reviewed by Bhagyalakshmi et al., 2007). Although this broad antimicrobial activity is not readily explained by the aforementioned activities, there is accumulating evidence that organosulfur compounds are able to interact with highly reactive or unshielded thiol moieties of target proteins or aminoacids (e.g., enzymes or cysteine, respectively) forming the corresponding thioallyl adducts (R-SCH_2_CHCH_2_), an event potentially life threatening for the cell. Components from garlic that may be involved on these thiol-disulphide exchange reactions are the thioallyl compounds (TACs) that include S-allyl cysteine (obtained by long-time organic fermentation of whole bulbs in ethanolic solutions), allyl thiosulfinates (e.g., allicin, obtained by crushing o macerating bulbs in water) and some products of allicin decomposition (allyl sulfides and ajoenes) (Lawson and Wang, 1993; Rabinkov et al., 1998; Gallwitz et al., 1999; Gupta and Porter, 2001; Pinto et al., 2006).

Garlic has been shown to have an effect in children suffering from giardiasis when used at 1:20 dilutions of aqueous extract in 2 doses per day for 3 days (Soffar and Mokhtar, 1991) and *in vitro* studies have shown that whole garlic extract and allyl alcohol have an effect on the physiology of *Giardia* trophozoites. These include cell surface rigidity, adhesion, flagellar activity, cytoskeletal components, and endomembrane elements (vesicles and vacuoles) while allyl mercaptan (AM, a TAC) preferentially induced vesicle distension and cell swelling, possibly related to a primary effect on membrane osmoregulation (Harris et al., 2000). Based on these observations it is likely that several garlic components do have different electivity in *G. duodenalis* trophozoites, however the mechanisms involved in their giardicidal activity are still undefined. Although this microaerophilic protozoan is devoid of glutathione as major non-protein thiol, *Giardia* possesses a plethora of cysteine-rich surface proteins, cysteine-type proteinases and other sulfur-containing intracellular proteins (Ali and Nozaki, 2007) that may be targets of TACs. In this work, we carried out an analysis on the structure-activity relationship (SAR), mode of action and efficacy in experimental giardiasis of representative TACs from garlic.

## Materials and methods

### Parasites

*G. duodenalis* trophozoites from the WB strain (ATCC # 30957) were axenically cultured in modified Diamond's TYI-S-33 medium (Keister, 1983) without bile at 37°C. Cell harvests were carried out by chilling flasks at the log phase of growth reached after 72–96 h routine subcultures. Detached trophozoites were either directly counted in a haemocytometer or washed 3 times with phosphate buffer saline (PBS) prior to counting and the cell number was adjusted as required in the assays.

### Garlic extracts and compounds

Fresh, aqueous garlic extract (AGE) was prepared by crushing peeled, chopped and weighed garlic bulbs (*A. sativum* L.) in distilled water (0.1 g/mL) at pH 6.5 using a mortar. After resting for 10 min, homogenate was filtered through a 0.45 μM pore size Millipore^TM^ membrane. The filtrate was recovered and used at the required concentrations. Regarding the garlic components the following compounds were used: alliin (S-allyl cysteine sulphoxide, ALI), diallyl sulfide (DAS) and dipropyl sulfide (DPS) from Fluka Chemika AG (Buchs, Switzerland); diallyl disulphide (DADS), dipropyl disulphide (DPDS), allyl methyl sulfide (AMS) and allyl mercaptan (AM) from Aldrich Chem. Co. (Milwaukee, WI, USA); methyl methanethiosulfonate (MMTS) and sodium p-hydroxymercuribenzoate (pHMB) from Sigma Chem. Co. (St. Louis, MO, USA); S-allyl-L-cysteine (SAC) from TCI (Tokyo Kasei Kogyo Co., Tokyo, Japan) and diallyl trisulfide (DATS) from MP Biomedicals Inc. (Solon, OH, USA). Allicin (diallyl thiosulfinate, ALC) was obtained by oxidation of DADS using the protocol originally reported by Lawson (Lawson, 1996; Argüello-García et al., 2010). In this, 1 g DADS was dissolved in 5 mL acetic acid under stirring on an ice bath. Hydrogen peroxide (1.5 mL, 30% v/v) was added stepwise and the reaction was allowed to proceed for 30 min. Then, the reaction was kept at 13°C for 20 min then it was placed on an ice bath for 5 h to minimize the content of remaining DADS. Reaction was stopped by adding 15 mL distilled water at pH 6.5 and it was extracted with 30 mL dichloromethane. Acetic acid was removed by 5 extractions with 5% (w/v) sodium bicarbonate (20 mL each) then by 3 extractions with distilled water (20 mL each). The solvent was left to evaporate until yellowish oil (ALC, yield of ~600–800 μL per assay) was obtained. For stabilization and storage of ALC, the oily product was resuspended in water at 1.5% (w/v) and kept in 1 mL aliquots at −70°C until analyzed and used. Under these conditions, ALC retained its full activity for up to 6 months. The identification of ALC was performed by Nuclear Magnetic Resonance (^13^C- and ^1^H-spectra) using a Jeol Eclipse^TM^ spectrometer with deuterated carbon trichloride (CDCl_3_) as solvent and tetramethylsilane as reference solution. Chemical shifts were recorded in ppm at 300 MHz in a Brucker system. Purity of ALC was determined by High Performance Liquid Chromatography (Waters model 996 coupled to a pump Waters model 600) using 4.6 × 250 mm Spherisorb^TM^ columns (10 μM particle size), mobile phase 30% methanol:70% water, flow rate 0.4 mL/min and UV detection at 254 nm, rendering an average time retention of 3.61 min and a mean purity of 90–92% for synthetic allicin.

### *In vitro* susceptibility assays

When complete TYI-S-33 medium (final cysteine concentration = 12.4 mM) was used, it was no older than 10 days to avoid cysteine oxidation. In these assays, 1 × 10^6^ trophozoites were exposed to increasing concentrations of AGE or TACs for 24 h at 37°C in 4.5 mL screw-capped vials. Positive controls included trophozoites treated with metronidazole at 3.0 and 5.9 μM (corresponding to its IC_50_ and MLC; Argüello-García et al., 2004) and as a negative control for the vehicle, 20 μL distilled water, pH 6.5 were included in all experiments to discard significant variabilities in standard drug susceptibility of the WB strain used in this study. Parasites were harvested and counted and trophozoite's viability was determined by physiological methods to determine the growth capacity of trophozoites after drug exposure (subculture in liquid medium SCLM)] or trophozoite and membrane integrity [direct cell count (DCC) and trypan blue exclusion (TBE), respectively]. Also, enzyme activity–based methods were used and these included fluorescein diacetate-propidium iodide staining (FDA-PI) that relies on the ability of cellular esterases in living cells to hydrolyse FDA into fluorescein while PI form complexes with nucleic acids of dead cells (Breeuwer et al., 1995); the 3-(4,5-dimethyl-2-thiazolyl)-2,5-diphenyl-2-H-tetrazolium bromide (MTT) reduction assay that is based on the crystallyzation of the formazan derivative of MTT in living cells by the action of cellular oxidoreductases (NAD(P)H-dependent dehydrogenases, reductases) (Liu et al., 1997). All these protocols have been described elsewhere (Argüello-García et al., 2004) except that DCC values were calculated from total cell numbers of compound-exposed trophozoites divided by the initial trophozoites' inoculum (1 × 10^6^) and TBE was performed by incubating PBS-washed parasites in a trypan blue solution (0.4% [w/v] final concentration) for 5 min at room temperature followed by microscopic count of “stained” (non-viable) parasites. As reference, the “gold standard” method (SCLM) consisted in the back growth of 1 × 10^5^ parasites for 48 h at 37°C after exposure to compounds and the interpolation of cell counts into a standard growth curve run in parallel using increasing number of parasites (5,000–250,000) non exposed to any drug.

In each method, the inhibitory concentration at 50% (IC_50_) and the minimal lethal concentration (MLC) corresponding to the lower concentration of compound that caused mortality in all cells were calculated using the concentration-viability curves in which the minimal-square method was used. Statistical significance between the data obtained in two or more experiments was determined by paired, two-tailed *t*-Student's test (Microsoft Excel^TM^).

### Electron microscopy studies

Trophozoites were exposed to TACs in two ways: under long (24 h, standard toxicity) and short (3 h, acute toxicity) incubations at 37°C using concentrations corresponding to the experimental MLC (as determined at 24 h) and twofold the MLC, respectively. For scanning electron microscopy, PBS-washed parasites were fixed with 2.5% glutaraldehyde in 0.1 M sodium cacodylate buffer, pH 7.2, dehydrated under increasing concentrations of ethanol, critical-point dried using a Samdri apparatus, gold coated in an ion sputtering device (JEOL-JFC-1100), and examined with a JEOL 35-C scanning electron microscope. In transmission electron microscopy (TEM) studies, washed cells were fixed with 2.5% glutaraldehyde and post-fixed with 1% osmium tetroxide in cacodylate buffer. Following sequential dehydration in ethanol, samples were embedded with epoxy resins. Thin sections were observed in a JEOL 100-SX transmission electron microscope.

### SAR analyses

The values of four electronic and two molecular transport (absorption prediction) descriptors have been previously determined for most TAC used herein (see Table 2 in Argüello-García et al., 2010). Briefly, the chemical structures of the ten compounds were constructed using Chemdraw Ultra 8.0 software (Cambridge soft Corp. Cambridge, MA, USA) and imported into SPARTAN'04 package (Wave function Inc. Irvine, CA, USA). The *ab initio* approach was used in this software to minimize structures and obtain equilibrium geometry *in vacuo* using the semi-empirical AM1 module. Thus, the minimal energy conformations (Boltzmann conformers) of each compound were obtained by default parameters in order to determine values for electronic properties that included HOMO (highest occupied molecular orbital, *E*_*HOMO*_) and LUMO (lowest unoccupied molecular orbital, *E*_*LUMO*_) eigen values, dipole moment (Debye) and total energy (*E*_*TOTAL*_). Regarding the two descriptors related to absorption prediction, the octanol/water partition coefficient (*log P*_*oct*_) values were retrieved from other studies while the molecular polar surface area (*PSA*) was calculated following the fragment-based contribution procedure. The relationship between the values of each of these molecular descriptors and the giardicidal activity (expressed as MLC values) were determined by multiple regression analysis (MLR) using the 16.0.18 version of Statgraphics Centurion XVI software.

### Thiol-modifying activity of TACs

The technique reported by Lawson and Wang (1993) was followed. In these assays trophozoites were collected from 60 mL flasks, washed 3 times with PBS and resuspended in 500 μL PBS containing a 1:12.5 dilution of a commercial mixture of protease inhibitors (Complete^TM^, Roche Diagnostics GmbH, Indianapolis, IN, USA) and lysed by sonication at 12 microns/60% amplitude (Ultrasonic apparatus Sonic UVX1130PB). Soluble proteins were recovered by centrifugation at 15,400 × g for 30 min at 4°C and protein concentration was determined by the Bradford technique. These samples were adjusted to a concentration of 1 mg/mL. Reactions were carried out in 1.5 mL microcentrifuge tubes with a mixture of 100 μg protein (100 μL) and NaH_2_PO_4_ 45 mM pH 7.0 mM (800 μL) to which increasing volumes of TACs stocks or a positive control with the thiol derivatizing agent MMTS prepared at 50 mM in the same buffer, were added and total volume was adjusted to 1 mL. After incubation for 10 min at 37°C, 100 μL of 1.5 mM dithionitrobenzoic acid (DTNB, Ellman's reagent) were added to each tube and following incubation for 10 min at room temperature the absorbances (yellow solutions) were recorded at 412 nm (Smart Spec 3000^TM^, Bio-Rad, Hercules, CA, USA). A calibration curve was obtained using increasing volumes (0–100 μL) of 15.5 mM cysteine instead protein extracts and the maximal blocking concentration (MBC) corresponding to a complete absence of thiol-DTNB complexes produced by TACs were calculated using the least-square method (Microsoft Excel^TM^).

### Proteolytic activity assays

Trophozoite lysates were prepared as mentioned above except that cell lysis was carried out by sonication in 1.5 mL microcentrifuge tubes under ice bath using eight 15-s pulses at 80% amplitude with resting periods of 30 s (Ultrasonic Processor mod. CV19). Protein concentration was adjusted to 2 mg/mL and aliquoted in 25 μL fractions that were incubated for 30 min room temperature with 2–5 μL stock solutions of representative TACs (ALC, DADS, and AM). The reaction mixtures were diluted in Laemmli's sample buffer, loaded in wells of 10% acrylamide-02% gelatin slab gels and these were electrophoretically analyzed using 80 V/4°C. After this, gels were incubated in a solution of triton X-100 2.5% (v/v) in water for 1 h and washed 3 times with distilled water. Bands with proteolytic activity were detected as follows: gels were incubated in acetate buffer pH 5.5 with 1 mM DTT for 16 h at 37°C under constant stirring, washed 3 times with distilled water and stained with Coomassie blue (BioRad) for 2 h at 37°C under constant stirring. Finally, gels were washed with distilled water and incubated with a solution of 5% acetic acid/40% methanol (v/v). Gel images were documented with a UVP High Performance Ultraviolet Transilluminator and software Launch DocItLS.

### Effect of representative TACs as possible inhibitors of the cytolytic effect of giardipain-1 on IEC6 cell monolayers

Based on the inhibitory concentrations of TACs over the activity displayed by the ≈25-kDa sized band containing giardipain-1 in zymograms, this protease purified by affinity chromartography from trophozoite lysates at a cytotoxic concentration (16 μg/mL; Ortega-Pierres et al., 2018) was pre-incubated for 30 min at room temperature in 200 μL DMEM medium alone or containing ALC (5.3 mM), DADS (275 mM), AM (514 mM), or E64 (20 μM). Additional assays were carried out using the MLCs of TACs (without giardipain-1) previously obtained in trophozoite cultures to monitor their toxicity. These solutions were added to monolayers of the intestinal cell line IEC6 grown in 1 cm^2^-microwells and incubated for 100 min at 37°C. After incubation, supernatants were discarded and monolayers were fixed with 2% paraformaldehyde in PBS. The effects of TACs and E64 on the cytolytic effect of giardipain-1 were evaluated by light microscopy using Nomarski optics (Axioskop 40, Zeiss).

### Flow cytometry analyses

The identification and quantitative determinations of trophozoites were carried out as follows: (a) apoptosis or necrosis were determined using anti-annexin V-fluorescein isothiocyanate (FITC) antibodies or PI as respective markers, (b) oxidative stress was determined by formation of reactive oxygen species (ROS) with the fluorescent ROS-complexing agent 5-(and 6)-carboxy 2′7′-dichlorodihydrofluorescein diacetate (H_2_DCFDA), and (c) determination of trophozoites cell cycle stages (G1, S, G2, and G2/M) after exposure to ALC or to a control drug (albendazole, ABZ) was carried out as previously described (Martínez-Espinosa et al., 2015). In all these assays 20,000 cells per sample were recorded.

### Efficacy of age and TACs in experimental giardiasis

Mongolian gerbils (*Meriones unguiculatus*) used in this study were kept at CINVESTAV's bioterium facilities under regulated conditions of temperature, humidity, and filtered air. Animals were fed *ad libitum* with rodent pellets (Purina^TM^) and water and monitored daily. The management of gerbils was performed according to the Mexican official norm NOM-062-ZOO-1999 for the production, care and use of laboratory animals (UPEAL-CINVESTAV). Eighty male Mongolian gerbils (*M. unguiculatus*) 6–10 weeks old, weighted 45–60 g and sentinel-checked gerbils free of intestinal parasites were kept in individual cages and distributed in 12 groups of 6 animals per treatment and experimental infections were performed as described elsewhere (Argüello-García and Ortega-Pierres, 1997). In brief, animals were inoculated intragastrically with 1 × 10^6^ *G. duodenalis* trophozoites in 500 μl sterile PBS *per os* using gavage under ether anesthesia. Control animals received 500 μl sterile PBS each. At day 10 post-infection (p.i.), gerbils were treated with AGE at single doses of 385, 770, 1,155, and 1,550 mg/kg or two doses of 770 mg/kg (days 10 and 11 p.i.) or a selected TAC (here ALC at 35, 70, 138, or 276 mg/kg at single dose). As additional control, one group of animals received MTZ (38.4 mg/kg at single or double dose). Only the higher, single doses of AGE (1.55 g/kg) and ALC (276 mg/kg) caused a noticeable irritation in intestinal epithelium as assessed by surgical examination of treated, non-infected animals (*n* = 1 per single dose). Treatment efficacy was evaluated by determining the presence or absence of trophozoites in intestinal contents of control and treated gerbils at day 13 post-infection. For this, gerbils were euthanized by an intraperitoneal injection of pentobarbital at a dose of 150 mg/kg to minimize stress and to reduce suffering, then a 10 cm-long section of intestine (from stomach ending, equivalent to nearly the entire small intestine) was excised after laparotomy, and it was longitudinally opened and rinsed in 20 mL PBS at 4°C for 20 min under shaking (720 rpm, Heidolph^TM^ rotatory shaker) to determine trophozoites number after parasite detachment from microvilli. Supernatant was collected and centrifuged at 750 × g for 10 min at 4°C, cell pellet was resuspended in 1 mL PBS and trophozoites were counted using a haemocytometer. Gerbils were scored as cured if no trophozoites were recovered from infected animals.

### Molecular modeling and docking studies

The three-dimensional protein structure of giardipain-1 was obtained from the sequence of GL50803_14019 deposited in GiardiaDB using the Swiss Model server (https://swissmodel.expasy.org/). To predict the ability of garlic TACs (ALC, DADS, and AM) as possible inhibitors of the protease activity of giardipain-1 by interaction with its catalytic moiety, *in silico* approaches of molecular docking were performed using the Swiss Dock web service (http://swissdock.ch). This platform delivers a set of 256 *viable* positions between rigid target (mGiardipain-1) and one flexible ligand (TACs) that are input in PDB and MOL2 formats, respectively. Of these predictions, only those involving a likely interaction of inhibitor with the catalytic Cys27 in giardipain-1 were selected and from these, the most favored docking corresponded to the lowest (i.e., negative value) Gibbs free energy (ΔG). In these analyses, the crystal structure of human Cathepsin B (PDB ID: 2PBH) was used as structure of reference. All structures and predictions obtained from Swiss Model, SwissDock and ZINC archives of TACs (www:// http://zinc.docking.org/) were visualized and edited using the UCSF chimera package v. 1.10.1

### Statistical analyses

In all experiments involving concentration-response curves the values obtained were adjusted with the least-square method to linearize calibration curves (Microsoft Excel 2010^TM^) and differences in mean ± SD values between two or more groups were assessed by the *t*-student test at a significance of *P* < 0.05.

## Results

### Efficacy and mode of action of age against *Giardia* trophozoites

**Figure 1:**
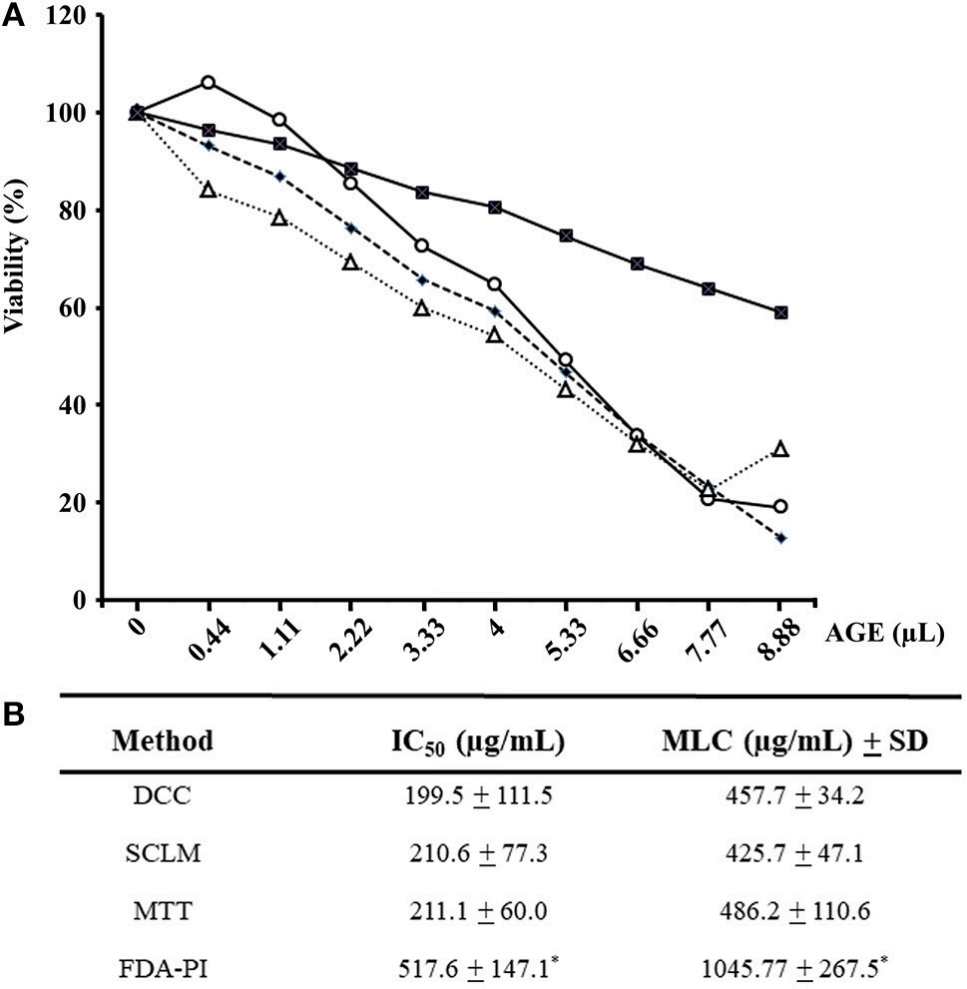
Evaluation of susceptibility of *G. duodenalis* trophozoites to aqueous garlic extract (AGE) using four methods. **(A)** Parasites (1 × 10^6^) were exposed to increasing quantities of 200 μg/mL AGE for 24 h and cellular viability was determined by four methods: subculture in liquid medium (open circles), FDA-PI staining (filled squares), direct cell count (filled diamonds), and MTT reduction (open triangles). Values correspond to results from three independent experiments. **(B)** The values of the inhibitory concentrations at 50% (IC_50_) and 100% (MLC) were calculated by the least square method. ^*^Significantly higher than any of the other methods at *P* < 0.05.

In all drug susceptibility assays, positive controls with 3.0 and 5.9 μM metronidazole inhibited trophozoite growth by 45–55% and more than 95%, respectively, as expected while distilled water pH 6.5 did not affect parasite's growth. In a first set of experiments, fresh AGEs were prepared and added at increasing concentrations to trophozoite cultures to assess parasite's viability by physiological (SCLM and DCC) and enzyme activity–based (FDA-PI and MTT) methods. It is worth mention that garlic components in the 0-400 μg/mL range produced a progressive decay (up to >80%) in the number of trophozoites with ability to either re-grow, maintain cellular integrity or reduce MTT; however even at 400 μg/mL AGE more than 60% of remaining cells were still positive for FDA-PI staining (Figure 1A). IC_50_ and MLC calculations confirmed a similar sensitivity of SCLM, DCC, and MTT assays and a significantly lower sensitivity of the FDA-PI method (Figure 1B), indicating that components from garlic extracts induce primary effects on trophozoite integrity concomitant to loss of oxidoreductase activities and a delayed detriment of esterase activities.

### SAR of TACs

To evaluate the giardicidal activity of the six garlic's TACs initially included and its relation to the compound structure the SCLM method was chosen considering its aforementioned higher sensitivity and from reports using well-known antigiardial compounds that include 5-nitroimidazoles and bencimidazoles (Argüello-García et al., 2004, 2009). These TACs have defined modifications in their chemical structure mainly involving increasing hydrocarbon chains with carbon-carbon double bonds and additions of sulfur and/or oxygen atoms or even α-aminoacidic groups thus rendering AM as the simplest and SAC as the most complex TAC evaluated herein. As shown in Table 1 the thioallyl group displayed in bold in each chemical formula and comparing experimental MLC values, the giardicidal efficacy of AM was drastically lowered (3.06 vs. 74.28 mM) by replacing the sulfur-attached Hydrogen by a methyl group as in AMS; however the replacement of this Hydrogen by an allyl group as in DAS partially recovers the efficacy in one order of magnitude (23.71 mM) below to that of AM. Interestingly the further addition of a second sulfur atom, as in DADS, recovers fully the efficacy to a comparable level of that displayed by AM (2.66 mM). Further the addition of a sulfur-attached oxygen atom, as displayed in ALC increased the giardicidal activity to sub-milimolar concentrations (0.6 mM) being ALC (allicin or diallyl thiosulfinate) the most effective TACs tested. In contrast, the presence of one α-amino carboxylic group (cysteine) in SAC decreased the efficacy of TACs to similar levels as that of AMS (69.1 vs. 74.28 mM). These assays indicated the importance of the dithiol moiety (-S-S-) to potentiate the activity of TACs against *G. duodenalis* trophozoites.

**Table 1:** Experimental MLC values of six TACs against *G. duodenalis* trophozoites determined by SCLM.

**Thioallyl compound**	**Chemical formula**	**IC_50_ (mM) ±SD**	**MLC (mM) ±SD**
Allyl Mercaptan (AM)	**H**_2_**C** = **CH-CH**_2_**-S**H	0.83 ± 0.2	3.06 ± 0.7
Allyl Methyl Sulfide (AMS)	**H**_2_**C** = **CH-CH**_2_**-S-**CH_3_	40.3 ± 3.5	74.28 ± 12.3
Diallyl Sulfide (DAS)	**H**_2_**C** = **CH-CH**_2_**-S**-CH_2_-CH = CH_2_	14.5 ± 2.3	23.71 ± 6.0
Diallyl Disulfide (DADS)	**H**_2_**C** = **CH-CH**_2_-S-S-CH_2_-CH = CH_2_	1.4 ± 0.6	2.66 ± 0.8
Allicin (ALC)	**H**_2_**C** = **CH-CH**_2_**-S**-S(O) -CH_2_-CH = CH_2_	0.32 ± 0.02	0.60 ± 0.1
S-Allyl Cysteine (SAC)	**H**_2_**C** = **CH-CH**_2_**-S**-CH_2_(NH_2_)-COOH	40.82 ± 6.3	69.10 ± 13.1

**Figure 2:**
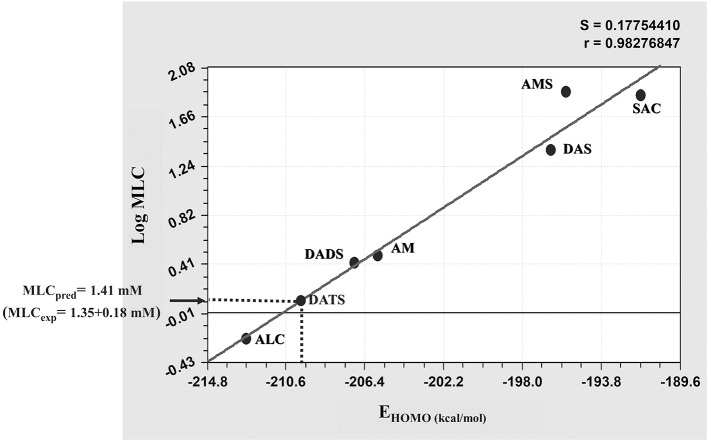
Linear regression model for the MLC-*E*_*HOMO*_ relationship of thioallyl compounds (TACs). The values of giardicidal activity (MLC) and the electronic descriptor E_HOMO_ of the six TACs included as “drivers” were processed by multiple linear regression and the value of E_HOMO_ calculated for the “test” compound diallyl trisulfide (DATS) was interpolated in the model (dotted line) to predict the MLC of this compound and to compare it with its experimetal MLC. Abbreviations of compound names are as indicated in section Materials and Methods.

In further analysis in which the activity of TACs were compared, an *in silico* approach based on MLR was used to determine if some of the four electronic (*E*_*TOTAL*_. *E*_*HOMO*_, *E*_*LUMO*_, dipole) and two molecular transport (logP_oct_, TPSA) descriptors calculated for these compounds (Supplementary Table 1) could be directly related to the giardicidal efficacy observed. In these analyses an additional TAC, diallyl trisulphide (DATS), was included as a “test” compound to evaluate the predictive value of the best structure-activity model raised. After these analyses, the molecular descriptor *E*_*HOMO*_ was the unique parameter associated to MLCs of the six TACs used as “drivers” to standardize the regression model. This latter rendered the lower variance (*S* = 0.177) and the highest regression coefficient (*R* = 0.9827) that were useful for this purpose (Figure 2). When the *E*_*HOMO*_ value calculated for DATS (−209.33 kcal/mol) was interpolated in this model a MLC = 1.41 mM was predicted. This value was closer to the experimental MLC = 1.35 ± 0.18 mM that was determined independently for this compound. Thus based on this approach we concluded that *E*_*HOMO*_ is a useful tool to predict the giardicidal activity of garlic's TACs.

### Differential thiol-disulphide exchange capacity of TACs

**Figure 3:**
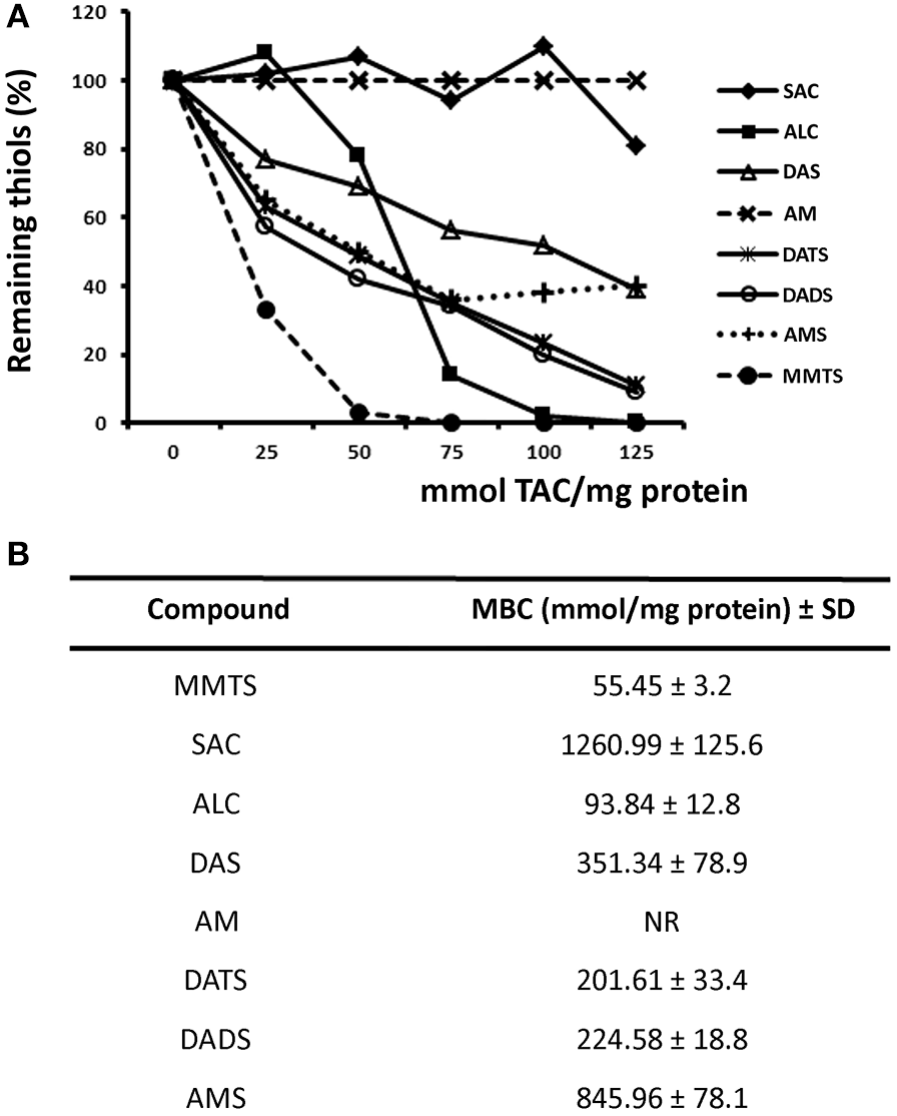
Inhibition of formation of thiol- dithionitrobenzoic acid (DTNB) complexes in protein extracts of *G. duodenalis* trophozoites by thioallyl compounds (TACs). **(A)** Parasite extracts (100 μg) were incubated with increasing concentrations of the indicated TACs then the thiol-derivatizing agent DTNB was added to the mixture. Samples without TAC were taken as controls with 100% thiols. Points represent the mean values of two independent experiments. Lines are as follows: S-allyl Cysteine (filled diamonds), Allicin (filled squares), Diallyl Sulfide (open triangles), Allyl Mercaptan (taches), Diallyl Trisulfide (astereisks), Diallyl Disulfide (open circles), Allyl-Methyl Sulfide (crosses), and Methyl Methanethiosulfonate (closed circles). **(B)** The values of the maximal blocking concentrations (MBC) for each TAC were calculated using a standard curve using cysteine.

Since TACs could modify thiol groups exposed in *G. duodenalis* trophozoite proteins into di-(thioallyl) derivatives thus hampering the further derivatization of these groups by DTNB, the blocking capacity of seven TACs was evaluated using parasite's total soluble extracts. The data obtained were in general compatible with their already known giardicidal activity, this is ALC was the most potent thiol-modifying compound tested (MBC = 93.8 mmol/mg protein) followed by DATS and DADS with MECs slightly higher to 200 mmol/mg. DAS had an intermediate efficacy (351.3 mmol/mg) and AMS and SAC exhibited a much lower efficacy (845.9 and 1260.9 mmol/mg, respectively) (Figure 3). Interestingly AM, in spite of its high giardicidal activity, it did not react with parasite extracts as noted by the increasing absorbance when higher concentrations of this compound were added and instead it formed complexes with DTNB. Indeed, the presence of increasing sulfur atoms and sulfur-attached oxygen influenced the higher efficacy of TACs to derivatize thiol groups exposed in giardial proteins.

### Differential inhibition of trophozoite's proteolytic activities by garlic TACs

**Figure 4:**
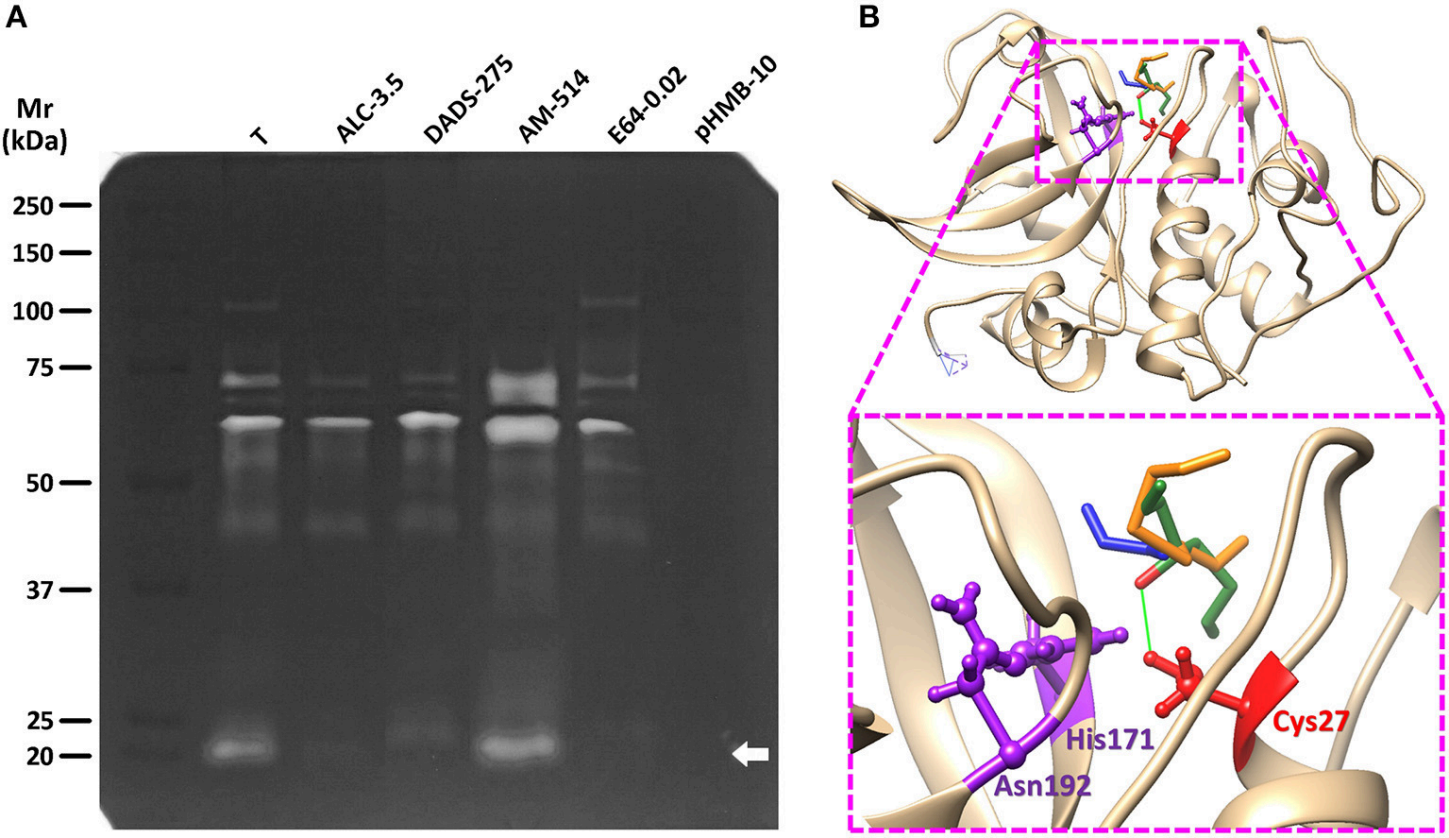
Effect of representative TACs on the proteolytic activity of *G. duodenalis* trophozoite lysates and docking with giardipain-1. **(A)** Trophozoite lysates (50 μg) were electrophoretically separated in 10% acrylamide-0.2% gelatin gels and cysteine protease activities were developed and detected by DTT treatment at pH 5.5 and Coomassie blue staining. Lanes in which extracts were pre-incubated with the three TACs tested (ALC, DADS, and AM) are indicated along with the concentration values (mM) used in each case. The thiol-active inhibitor pHMB (10 mM) and the cathepsin B-specific inhibitor E64 (20 μM) were included as controls. Molecular weight standards and its relative mobility (Mr) are as indicated at the left. The arrow points to the band containing the cysteine protease giardipain-1. **(B)** The most favored docking position of representative TACs (in stick conformation, green: ALC, orange: DADS, blue: AM) at the proximity of the catalytic Cys27 of giardipain-1 in which the Oxygen atom of ALC is predicted to form a bridge with the Hydrogen G1 of Cys27 at a distance of 2.425 Å (green line). The three catalytic residues (Cys26, His171, and Asn192) are represented in ball-and-stick conformation.

Considering the thiol-modifying activity of TACs on trophozoite protein extracts, three representative compounds showing high (ALC), intermediate (DADS), and low (AM) activities were chosen to evaluate their inhibitory effect on cysteine protease activities in trophozoite lysates. Zymogram analysis showed that lysates displayed several bands of proteolysis (mostly between 25 and 110 kDa; Figure 4A) of which bands of 75, 60, and 25 kDa were the most prominent. Some of these activities (110, 60, 75, and 25 kDa) were strongly inhibited by 3.5 mM ALC while DADS at a much higher concentration (275 mM) could diminish similar activities except for the one at 60 kDa. Intriguingly AM produced a significant increase of activity in bands of 75, 60, and 25 kDa though this might reflect its lack of reactivity toward protein –SH groups and its “cooperative” activity with DTT. As expected, a full inhibition of all proteolytic bands was observed with the unspecific thiol-active inhibitor pHMB at 10 mM. On the other hand the typical cathepsin B protease inhibitor E64 displayed a selective inhibition on the 25 kDa band that contains the processed form of the cathepsin B-like protease named as giardipain-1 (Ortega-Pierres et al., 2018). By molecular modeling and docking approaches, the higher inhibitory activity of ALC toward proteases like giardipain-1 could be at least partially explained by the predicted ability of its Oxygen atom to form an electron bridge with the Hydrogen G3 of the catalytic Cys26 (Figure 4B) thereby interfering with protease activity. This is reinforced by the similar entropy values predicted for these TACs (ΔG_ALC_ = −5.561 kCal/mol; ΔG_DADS_ = −6.093 kCal/mol; ΔG_AM_ = −5.554 kCal/Mol). Taken together, these data are concurrent with the notion that garlic TACs target several types of cysteine proteases in *G. duodenalis* trophozoites.

### Inhibition of cytopathic effects of mgiardipain-1 by representative garlic TACs

**Figure 5:**
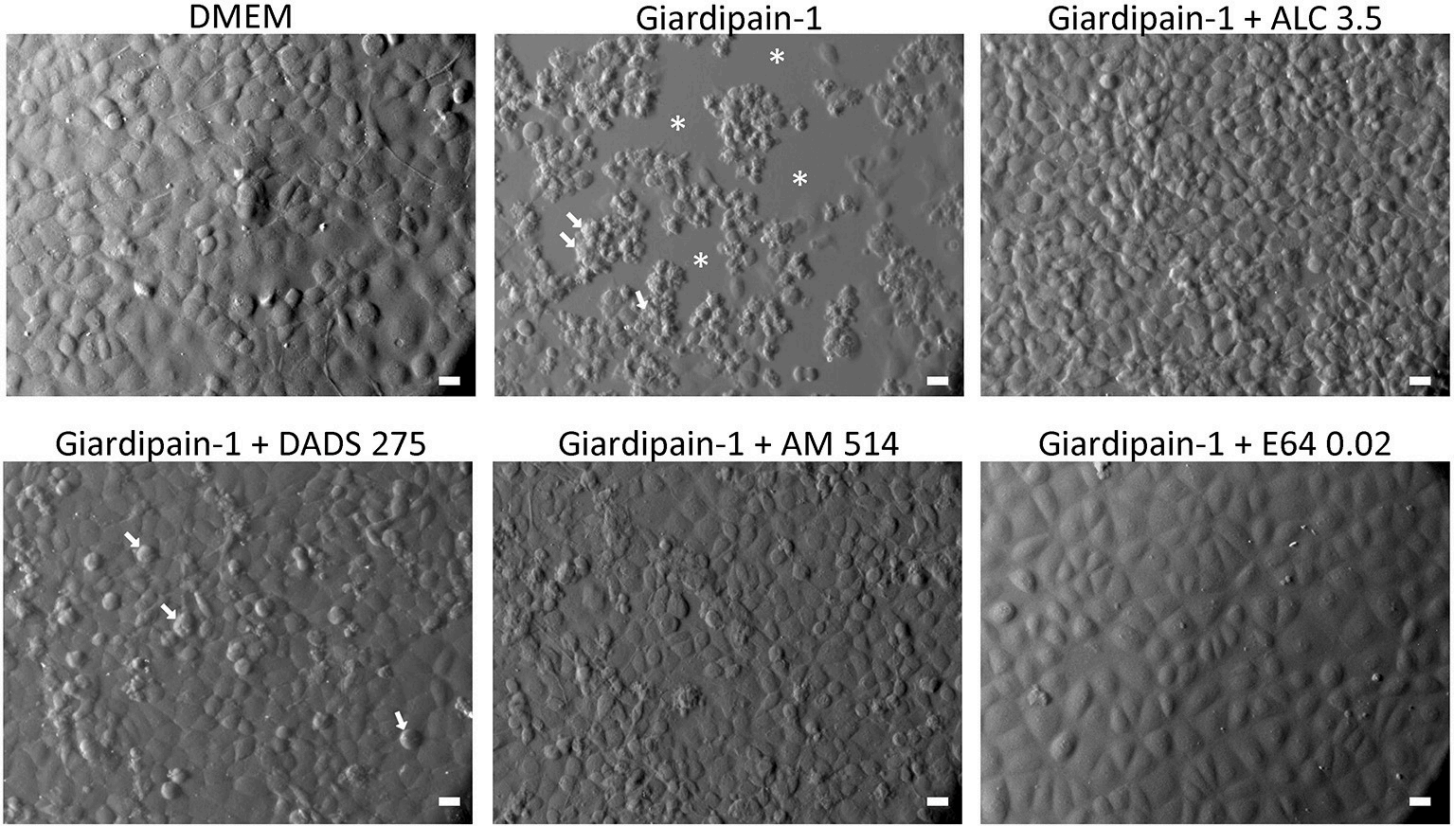
Effect of garlic TACs on the cytolytic damage caused by giardipain-1 in epithelial IEC6 cell monolayers. Confluent IEC6 cells were exposed for 100 min at 37°C to affinity chromatography-purified giardipain-1 alone or pre-incubated with the concentrations indicated (in mM) of representative TACs that caused inhibition of its protease activity in zymograms and monolayers were observed by Nomarski optics. Asterisks denote zones of monolayer destruction and arrows point to cells undergoing apoptotic death as indicated by membrane blebbing, shrinkage, and shape degeneration. Bars: 20 μm.

Once the concentrations of ALC, DADS and E64 used in zymograms were shown to inhibit a specific cysteine protease of *G. duodenalis* trophozoites, namely giardipain-1 (≈25 kDa), next it was assessed if these compounds could also block the already reported cytopathic effects of giardipain-1 as are shrinkage, detachment from surface, rounding and/or profuse vacuolisation (Ortega-Pierres et al., 2018). As shown in Figure 5, giardipain-1 causes a strong destruction of monolayers beginning with cell shrinkage and profuse blebbing (arrows) and the subsequent appearance of zones with absence of cells reflecting extensive destruction (asterisks) that are consistent with the pro-apoptotic damage reported for this secreted cysteine protease (Ortega-Pierres et al., 2018). However, the pre-incubation of giardipain-1 with ALC and AM noticeably blocked these effects while DADS reduced the frequency of cells displaying apoptotic damage. In general, these observations support a correlation between the inhibitory ability of garlic TACs on the proteolytic activity of giardipain-1 and the damage blockade exerted by these compounds. As expected, the potent cathepsin B inhibitor E64 efficiently inhibited the giardipain-1 induced damage in IEC6 cell monolayers. Of interest, with the exception of DADS concentration used in zymography analyses (275 mM) that affected some cells, none concentrations of TACs tested either at protease-inhibiting level or at MLCs that kill trophozoites in culture, were harmful to cell monolayers (Supplementary Figure 1). These results suggest that TAC's have a pharmacologically favorable and selective toxicity against trophozoites over epithelial cells.

Based on these comparative analyses, ALC was the garlic's derivative with the highest antigiardial activity, with the lower HOMO value, with the highest thiol-modifying activity and with the higher cysteine protease inhibitory capacity. Due to these properties, ALC was chosen in further studies to analyse its mechanism of action over trophozoites at different levels.

### Allicin has a primary cytolytic mode of action like age against *Giardia* trophozoites

**Figure 6:**
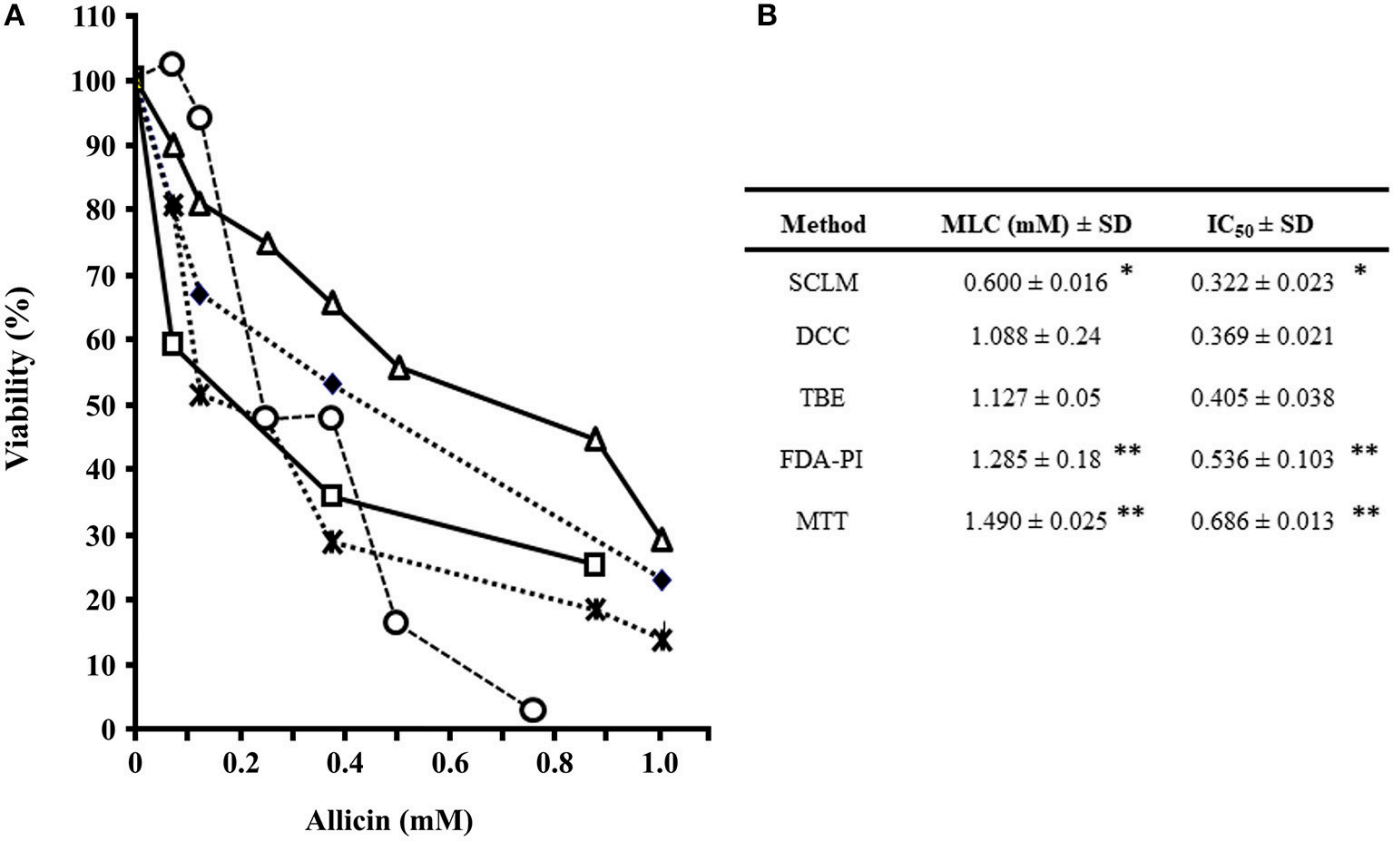
Evaluation of suceptibility of *G. duodenalis* trophozoites to allicin using five methods. **(A)** Parasites (1 × 10^6^) were exposed to increasing quantities of 2.5% ALC for 24 h and cellular viability was determined by several methods as previously described (Argüello-García et al., 2004). Values correspond to results from three independent experiments. Lines are as follows: subculture in liquid medium (open circles), fluorescein diacetate-propidium iodide staining (FDA-PI) staining (filled diamonds), direct cell count (taches), 3-(4,5-dimethyl-2-thiazolyl)-2,5-diphenyl-2-H-tetrazolium bromide (MTT) reduction (open triangles) and trypan blue exclusion (open squares). **(B)** The values of IC_50_ and MLC were calculated by the least square method. ^*^Significantly lower than any of the other methods at *P* < 0.05. ^**^Significantly higher than SCLM, DCC, and TBE (*P* < 0.05).

Since AGE had previously showed a detrimental effect on trophozoite's integrity followed by a decreased functionality of metabolic enzymes, we tested if ALC could have an effect as observed with whole garlic extracts. In these assays, the TBE protocol was also included in order to assess whether the integrity of trophozoite's membrane is primarily targeted by ALC. As expected, SCLM was the most sensitive method where cell viability completely decreased at concentrations below 0.8 mM ALC while all other methods still displayed viability rates of >20% (Figure 6A). Thus parasite replication was initially suppressed at 600 μM ALC; the other two physiological methods (DCC and TBE) showed a concomitant decrease of viability (1.08 and 1.12 mM, respectively) and the two enzyme-based assays (FDA-PI and MTT) showed the lowest sensitivity (Figure 6B), suggesting that ALC primarily affected the cellular replication by impairing membrane permeability hence cellular integrity, with a secondary effect on the activity of metabolic enzymes as oxidoreductases and diesterases. When compare the AGE with ALC the latter had a primary cytolytic mechanism on trophozoites albeit oxidoreductases (MTT) were affected more lately by ALC.

### Electron microscopy analyses of the damage caused by allicin on *Giardia* trophozoites

**Figure 7:**
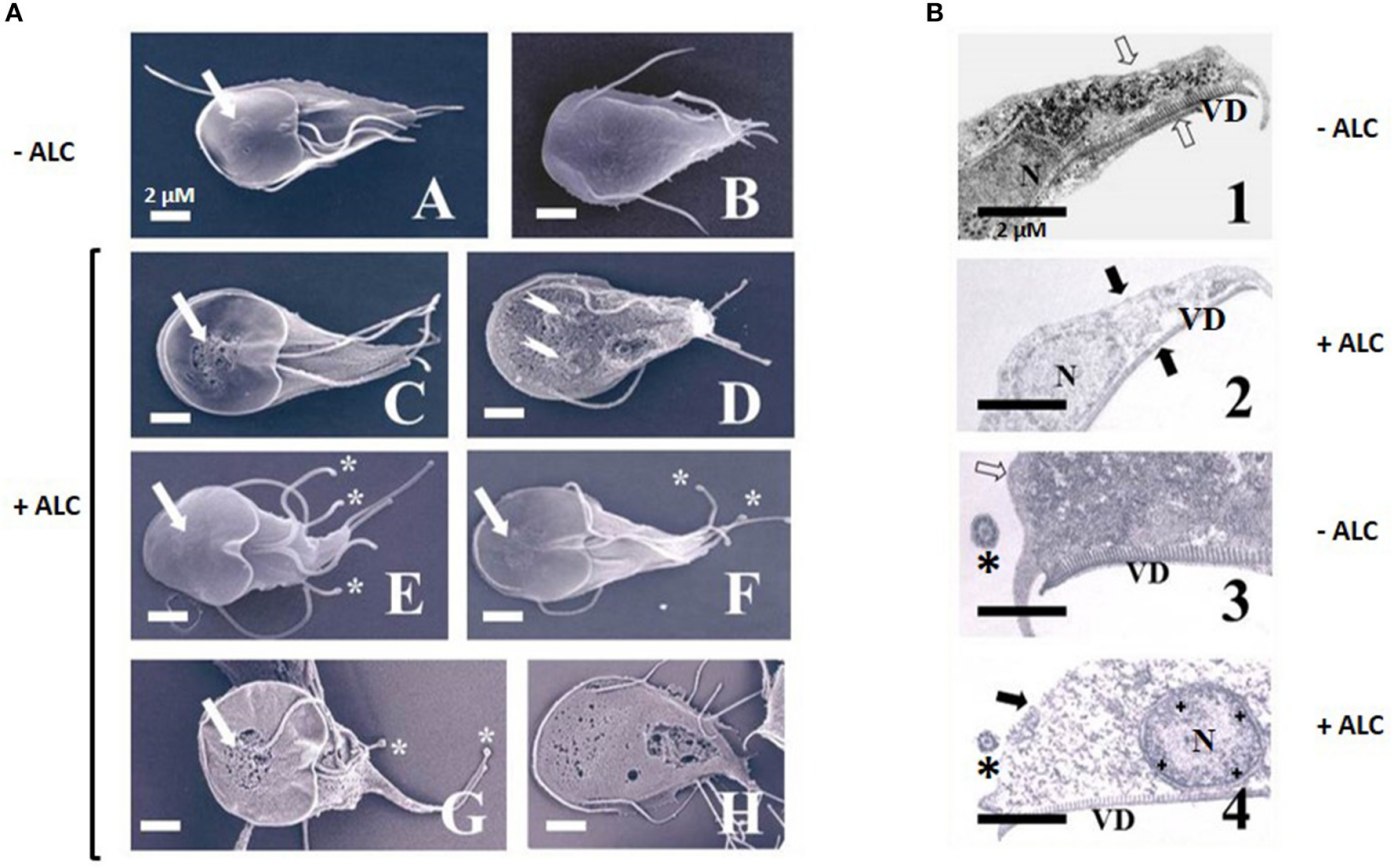
Analysis by scanning **(A)** and transmission **(B)** electron microscopy of *G. duodenalis* trophozoites exposed to allicin. (A) Parasites (1 × 10^6^) were either not exposed (A,B) or exposed to 600 μM ALC (C–F) for 24 h or 1.2 mM ALC for 3 h (G,H) at 37°C. Trophozoites are shown in ventral (A,C, E–G) and dorsal (B,D,H) views. The higher roughness of cell surface in images C–H as compared to A,B, except over the ventral disk, are indicative of a degree of membrane blebbing. Arrows denote the so-called “bared area” of the ventral disk and asterisks point to tip rounding at flagella. **(B)** Parasites not exposed (1, 3) and exposed to 600 μM ALC (2, 4) for 24 h at 37°C. Trophozoites are shown in longitudinal (1, 2) and cross-sections (3, 4). Arrows indicate the plasma membranes, asterisks show flagellar axonemes and crosses show inner peripheral areas of nucleus with chromatin condensation. N, nuclei; VD, ventral disk. Bars correspond to the indicated size.

To further analyse the effect of ALC on trophozoites a standard toxicity (24 h-exposure to 1 MLC) and an acute (3 h-exposure to 2 MLC) procedures were used. By SEM, control trophozoites exhibited the typical half a pear-shaped morphology with four pairs of flagella and a relatively smooth surface at ventral (Figure 7A-A) and dorsal (Figure 7A-B) planes. During standard toxicity, ALC provoked the appearance of multiple “holes” particularly in the so-called *bare area* of the ventral disk, which retained its normal contour (Figure 7A-C, arrow) along to the presence of small protrusions in flagella. The dorsal surface also presented a rough texture that resembled a membrane blebbing process with profuse holes and even nuclear membranes had severe damage (Figure 7A-D, arrows). In other cells the contour of the ventral disk appeared as less rigid and flagella displayed rounded tips (Figures 7A-E,F, asterisks). In parasites under acute ALC toxicity, the bare area showed severe damage in which cell surface presented multiple protrusions (blebbing) and flagella also showed rounded tips (Figure 7A-G, arrow and asterisks) while dorsal surface showed holes with a greater size and damaged areas that allowed the release of cytoplasmic material (Figure 7A-H). Of note, cell shrinkage was not a typical feature because ALC-treated cells did not display a contracted size (compare images Figure 7A and Figure 7B with the corresponding ventral or dorsal views).

At the intracellular level, in control cells the TEM analyses showed integrity of plasma membrane, peripheral vacuoles, nuclei and cytoskeletal elements as ventral disk microtubules and associated microribbons and flagellar axonemes with the typical 9+2 arrangement (Figures 7B-1,3). Regardless of the standard or acute toxicity protocols, ALC treatment leads to a complete loss of electron density of the intracellular elements and the integrity of plasma membrane, a diffuse destruction of cytoplasmic content rendering a granulose appearance and even nuclear envelopes seemed to be compromised (Figures 7B-2,4). In this context, a profuse condensation of chromatin at the inner periphery was seen (Figure 7B-4). As a consequence, a profuse vacuolisation pattern that occurs frequently with antigiardial drugs was not observed with ALC. Under these conditions, all cytoskeletal elements at ventral disk, cell periphery and flagella remained structurally unaltered and also cell shrinkage was not observed by SEM. These observations reinforced the presence of a cytolytic effect of ALC on *Giardia* trophozoites that promoted alterations at cellular (and even nuclear) membrane(s)'s integrity associated to further destruction of intracellular structures. To gain more insights on the ALC-mediated damage, we tested whether programmed cell death, oxidative stress and cell cycle arrest could be induced by ALC and then we compared these data with those obtained using ABZ which effects under similar conditions have been already described (Martínez-Espinosa et al., 2015).

### Pro-apoptotic, non-prooxidant and G2-arresting effect of allicin on trophozoites

**Figure 8:**
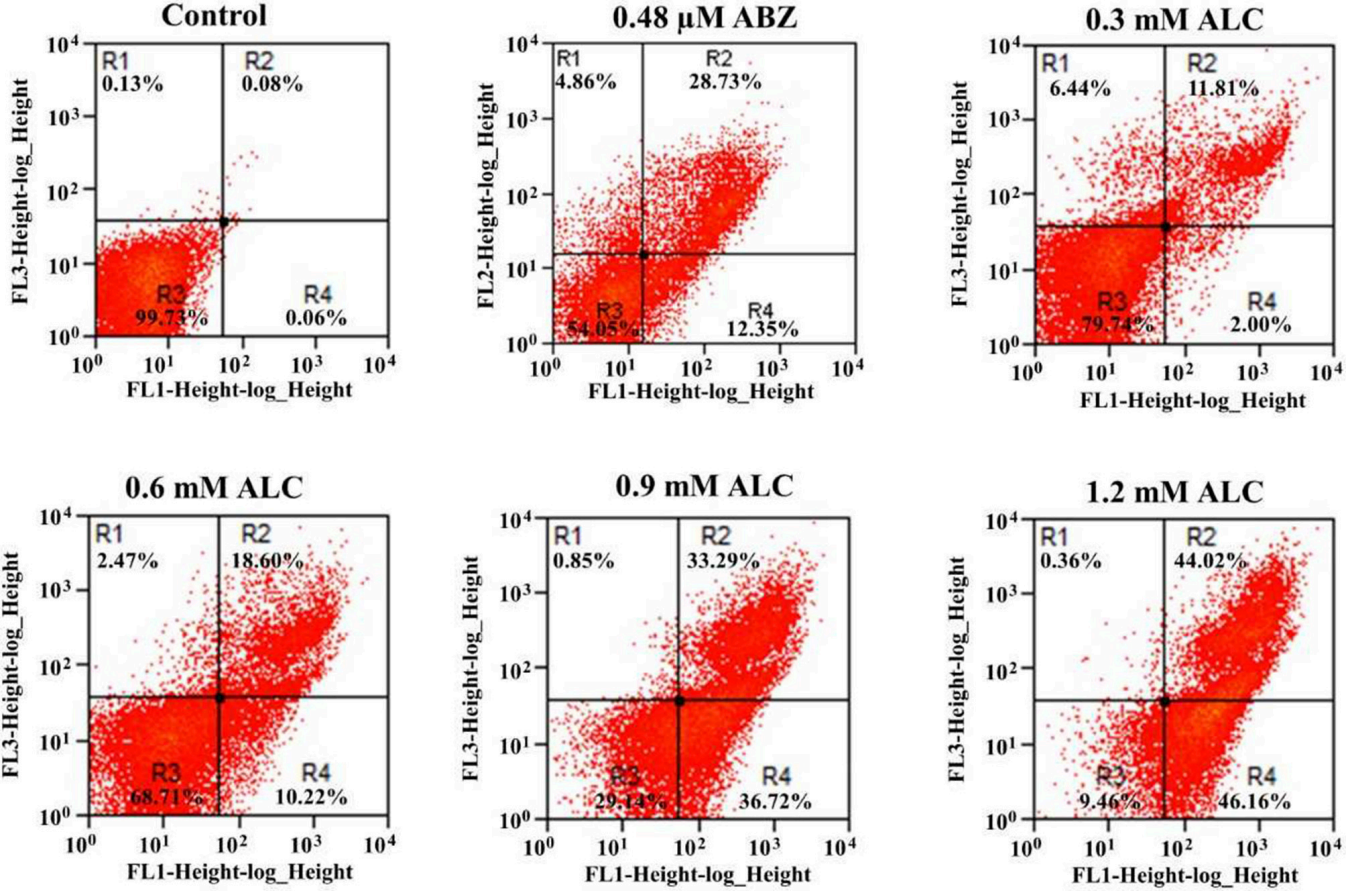
Allicin promotes apoptosis-like death in *G. duodenalis* trophozoites. Parasites (1 × 10^6^) were exposed to 1.0% double distilled water pH 6.5 (Control) or the indicated concentrations of ALC for 24 h at 37°C then stained with annexin V-PI and processed by flow cytometry. Data shown in the figure are as follows: quadrant R1 are cells positive for necrosis (PI+); quadrant R2 are cells positive for late apoptosis (annexin V+/PI+); quadrant R3 are cells negative for both markers and quadrant R4 are cells positive for early apoptosis (annexin V+). As a positive control, trophozoites were exposed to the indicated cytotoxic concentration of albendazole (ABZ). Percentages of total cells are indicated within each quadrant. The dot plots are representative of two independent experiments.

Based on the fact that ALC caused cytolysis in *G. duodenalis* trophozoites, it was of interest to determine the way by which parasites were killed by this TAC. To assess if ALC at increasing concentrations induces apoptotic or necrotic cell death in *G. duodenalis* trophozoites, anti-annexin V antibodies coupled to FITC and PI were used as markers of apoptosis and necrosis, respectively. In these assays, ABZ was used as control because of its known ability to induce apoptosis-like death in *G. duodenalis* (Martínez-Espinosa et al., 2015). As can be seen in a representative experiment shown in Figure 8, upon exposure to ALC at MLC (0.6 mM) 10.2% of trophozoites were at early apoptosis and 18.6% displayed late apoptosis and by increasing ALC concentration up to twice the MLC (i.e., 1.2 mM) three-times more cells were at apoptosis (46.6% in early and 44.0% in late stage, respectively). In all samples the percentages of cells in necrosis was always <2.5%. Control trophozoites exposed to ABZ even at 1.5 times its MLC (0.48 μM) only induced 41.0% cells in apoptosis and 5% cells were necrotic. These results indicated that ALC leads to a marked apoptosis-like cell death in trophozoites.

**Figure 9:**
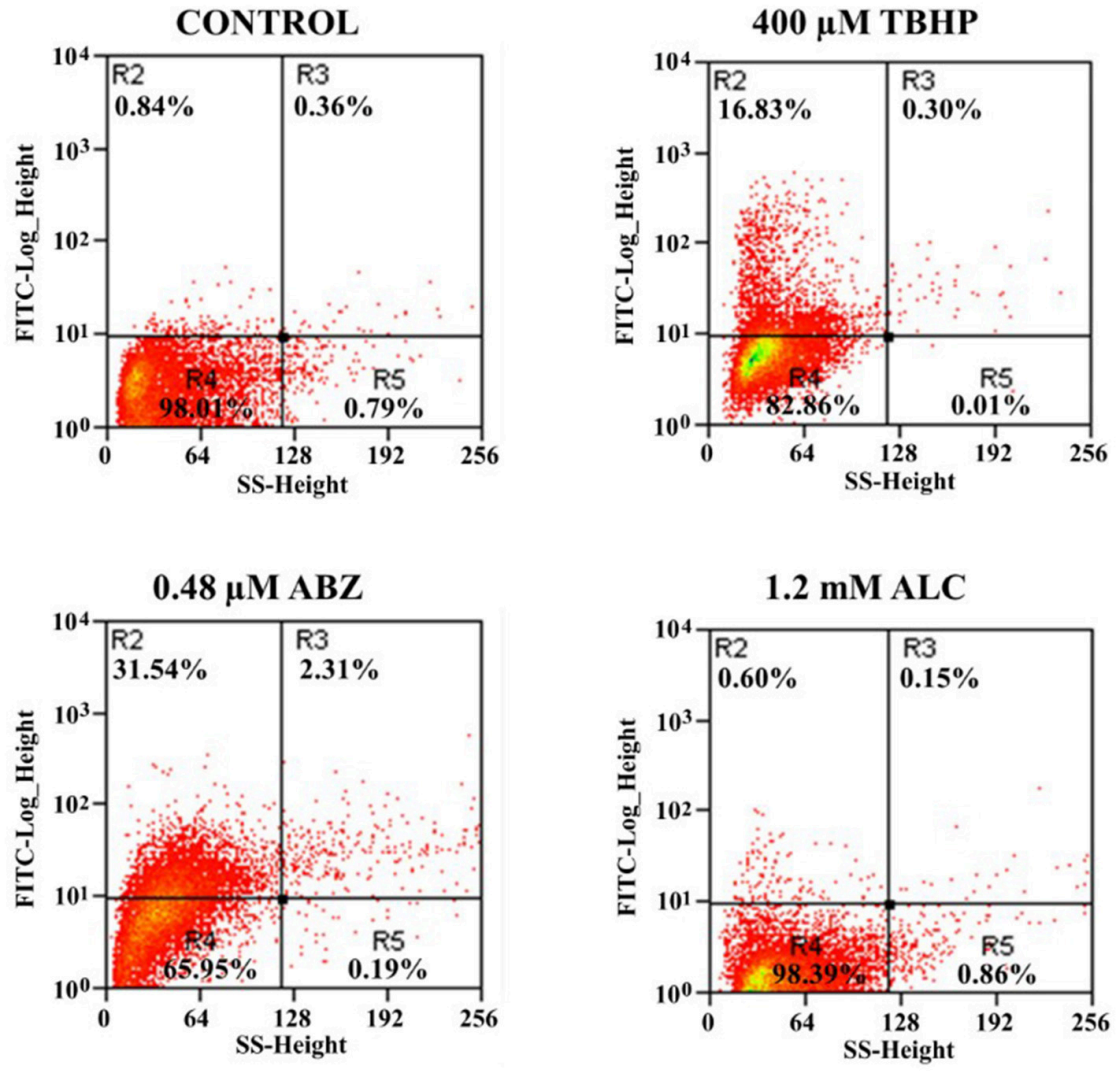
Allicin does not cause reactive oxygen species (ROS) formation in *G. duodenalis* trophozoites. Parasites (1 × 10^6^) were exposed to 1.0% double distilled water pH 6.5 (Control) or the indicated concentrations of ALC, tert-butyl hydroperoxide (TBHP, positive control) or prooxidant ABZ for 24 h at 37°C then incubated with the ROS-monitoring agent H_2_DCFDH and processed by flow cytometry. Data shown in the figure are as follows: quadrants R2-R3 are cells positive for ROS and quadrants R4-R5 are cells negative for ROS. Percentages of total cells are indicated within each quadrant. The dot plots are representative of two independent experiments.

Next, we tested if apoptosis-like death induced by ALC was associated with oxidative stress. In this, ROS were monitored in ALC-exposed trophozoites using the fluorescent tracer H_2_DCFDA. Figure 9 shows representative results in which, as compared to controls, only 0.75% cells were positive for ROS even using twice the MLC of ALC (1.2 mM) while using the control drug ABZ up to 33.85% of trophozoites displayed ROS formation as previously reported (Martínez-Espinosa et al., 2015). With the well-known ROS inducer tert-butyl hydroperoxide (TBHP) up to 17.1% cells were ROS-positive. These results demonstrated that ALC does not induce oxidative stress in *G. duodenalis* trophozoites as a death-associated mechanism.

**Figure 10:**
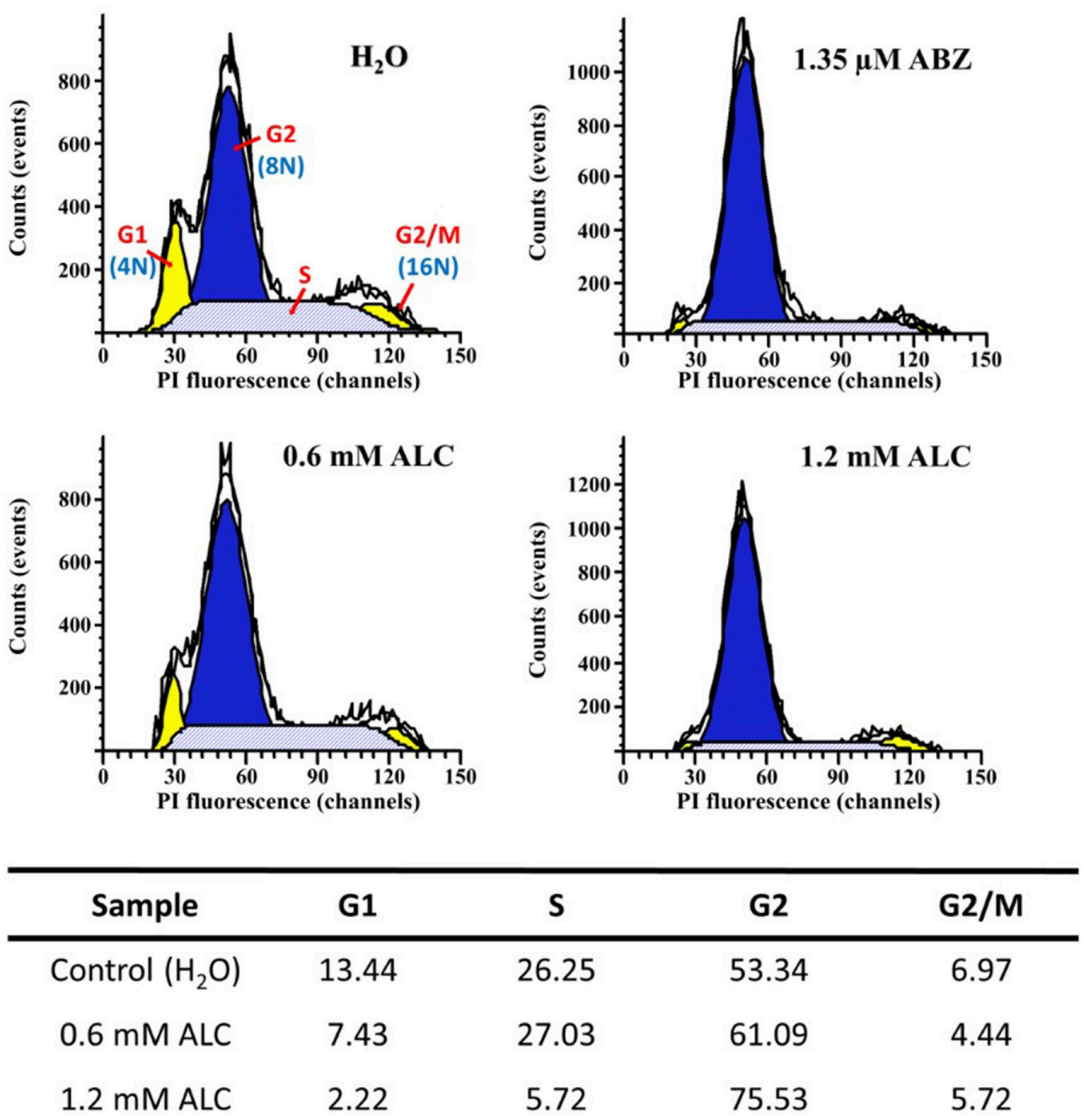
Allicin partially arrests *G. duodenalis* trophozoites at G2 phase. Parasites (1 × 10^6^) were exposed to 1.0% double distilled water pH 6.5 (Control) or the indicated concentrations of ABZ (positive control for cell cycle arrest) or ALC for 24 h at 37°C. Nuclei of permeabilised and RNAse A-treated cells were stained with PI and processed by flow cytometry. The areas under curve (AUC) corresponding to G1, S, G2, or G2/M cell cycle phases are indicated in the control histogram. Percentages of cells within each AUC are shown in the table *at the bottom* of histograms. Data are representative of two independent experiments.

Further flow cytometry analyses to determine the point(s) at which ALC interrupts cell cycle progression of vegetative trophozoites (Figure 10) showed that in control cells most of these were at G2 phase (53.3%) followed by a subpopulation at S phase (26.2%) whereas a lower proportion of cells were at G1 phase (13.4%) and the boundary G2/M contained 6.9% of cells. When trophozoite cultures were exposed to MLC and twice the MLC of ALC (0.6 mM and 1.2 mM) there were some changes: a marked *decrease* in G1 subpopulation with both ALC concentrations (7.4 and 2.2%, respectively), a significant *decrease* in S subpopulation with 1.2 mM ALC (5.7%) and an important *increase* in cells at G2 phase (61.0 and 75.3%, respectively). Interestingly, cells at G2/M phase did not significantly change (4.4 and 5.7%). As a reference, 1.35 μM ABZ partially decreased cells S at phase (22.1%) and partially arrested cells at G2 phase (69.9%) as previously reported (Martínez-Espinosa et al., 2015). All these data suggest that cytotoxic ALC concentrations allow an exit of G1 and S phases but most parasites were partially arrested at the G2 phase.

### Antiparasitic efficacy of AGE and ALC in experimental giardiasis

**Figure 11:**
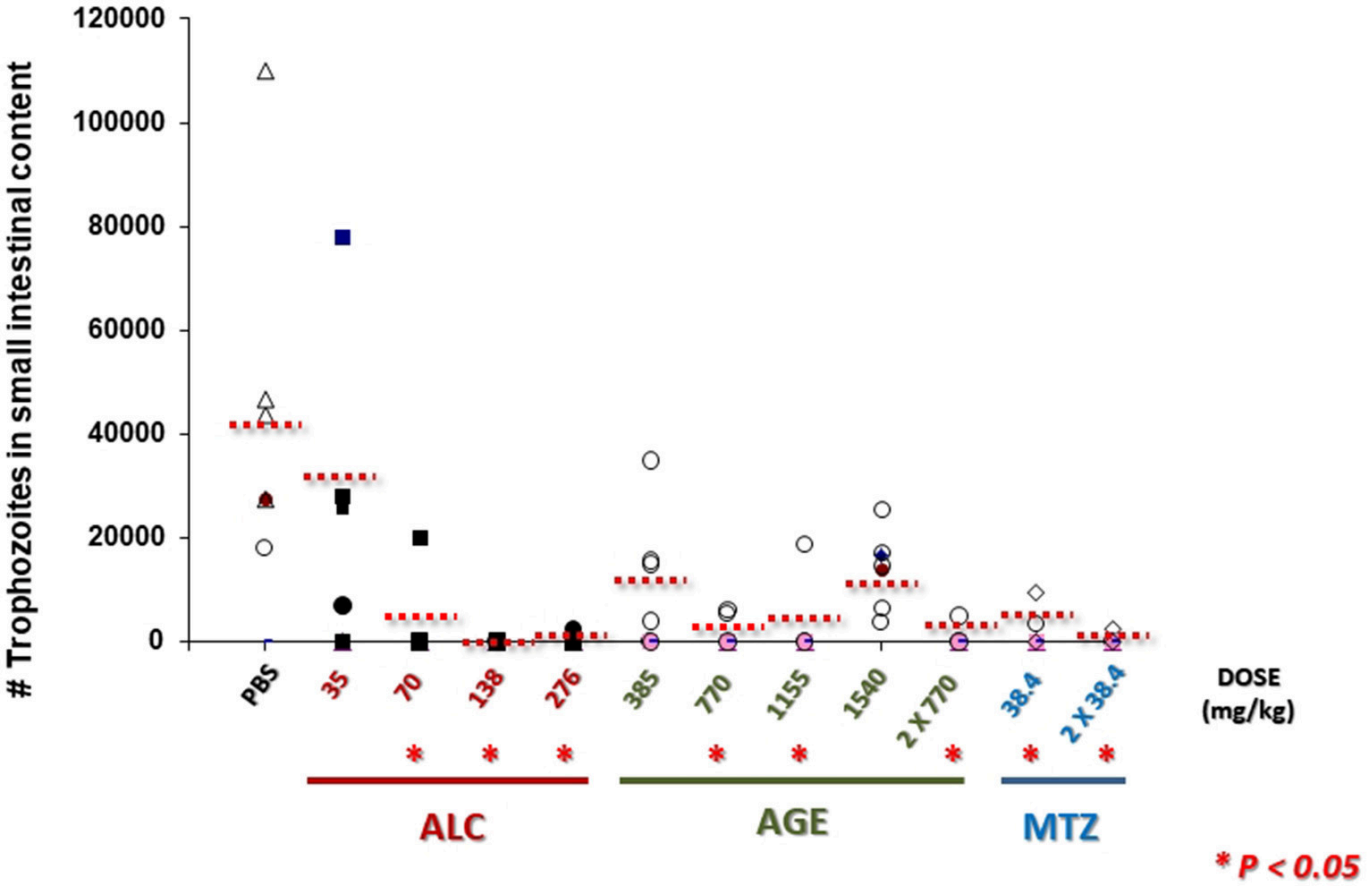
AGE and allicin diminish and eliminate *G. duodenalis* trophozoites in small intestines of infected gerbils. Male *M. unguiculatus* were intragastrically inoculated with 1 × 10^6^ trophozoites *per os*. At day 10 p.i. gerbils were treated with AGE (single or doubled doses), ALC at single doses or metronidazole (MTZ) at single or double dose as indicated. At day 13 p.i. trophozoites in intestinal contents (10 cm-long sections) were counted. Punctuated lines indicate the mean values of intestinal trophozoites for each group. Asterisks indicate counts significantly lower than control group (PBS) at *P* < *0.05*.

The pre-clinic and potential therapeutic effectiveness of AGE and ALC to treat giardiasis was evaluated in this work using the well-established model of experimental infections in Mongolian gerbils (Belosevic et al., 1982; Argüello-García and Ortega-Pierres, 1997). As shown in Figure 11, AGE-treated animals exhibited a significant reduction in trophozoite numbers with all doses tested as compared to PBS-treated, control gerbils. However only partial cure rates were obtained with 385 (2 out of 6 gerbils), 770 (3 out of 6), 1,155 (5 out of 6) and 770 mg/kg twice (5 out of 6) while none gerbil was cured with 1,515 mg/kg. Interestingly in ALC-treated animals, a progressive decrease in parasite numbers was observed upon increasing doses and cure rates increased as follows: 3 out of 6 gerbils with 35 mg/kg, 5 out of 6 with 70 mg/kg, 6 out of 6 with 138 mg/kg and 5 out of 6 with 276 mg/kg. These two latter doses were even more effective than the control drug MTZ that cured 3 out of 6 gerbils with 36 mg/kg and 5 out of 6 animals at twice dose.

All these data showed that ALC at increasing single doses had a dose-dependent and consistent giardicidal effect that caused a complete elimination of trophozoites in infected animals and a partially effective action using AGE. In spite that ALC is a major thiosulfinate in garlic extracts (Lawson, 1996) these differences are likely attributed to a distinct availability of ALC at intestinal lumen of infected animals to reach its targets in resident trophozoites.

## Discussion

Garlic has widely proven to be a rich source of nutraceutical compounds (Bhagyalakshmi et al., 2007) that are mostly organosulfur compounds as TACs. Indeed, garlic's TACs have a double benefit, i.e., health promoting and illness fighting, upon dietary or supplementary consumption. In recent years TACs contained in aqueous (e.g., ALC), ethanol (e.g., SAC) or oily (e.g., allyl sulfides as DATS, DADS, DAS, AMS, AM) garlic extracts have been studied in relation to the treatment of infectious diseases because of their direct effects over a plethora of pathogens (Ankri and Mirelman, 1999; D'Souza et al., 2017). In this work, the seven TACs mentioned above were evaluated in *G. duodenalis* trophozoites regarding its cytotoxic mechanism and therapeutic potential in experimental giardiasis.

Our results showed that the AGE's MLC as determined in this work (425 ± 47 μg/mL) is slightly higher than the value of 300 μg/mL previously determined (Harris et al., 2000). It is tempting to speculate that the use of two parasite strains (WB vs. Portland-1) along to different garlic sources (fresh cloves macerated in water vs. freeze-dried powder vortexed in culture medium) may account for the differences obtained in both studies. In addition, values of MLCs of four similar TACs used in our study and in another work (AM, AMS, DAS, and DADS) differed quantitatively, albeit AM and DADS were in both cases more active against trophozoites than either DAS or AMS. When its effect was compared to the one observed against other pathogens, AGE had a lower effectivity against enteral bacteria e.g., *Helicobacter pylori* (MLC: from 5 to 25–400 mg/mL) (Cellini et al., 1996; Moghadam et al., 2014) or even anaerobic protozoa as *Trichomonas gallinae* (MLC: 75 mg/mL) (Seddiek et al., 2014) thus highlighting the importance of the conspicuous repertoire of thiol-containing proteins and the dependence on cysteine import and metabolism in *G. duodenalis* (Ali and Nozaki, 2007) concomitant to its higher susceptibility to garlic constituents.

The use of physiological and enzyme activity based protocols were useful to determine that AGE had a cytolytic mechanism (i.e., TBE and DCC assays displayed high sensitivity) concomitant to an impaired oxidoreductase capacity as assessed by MTT assays and a further impairment of diesterase activity in trophozoites as shown by the lower sensitivity of FDA-PI assays. The effect of garlic components on the integrity of cell membranes were in agreement with a previous report (Harris et al., 2000) that showed a collapse in the electrochemical potential of trophozoite plasma membranes exposed to AGE and allyl alcohol. In this context, the abundance of cysteine (i.e., thiol)-rich proteins in *Giardia* surface proteins (Adam, 2001; Pinto et al., 2006; Ali and Nozaki, 2007) play a direct role as primary targets of garlic constituents, especially organosulfur compounds as TACs. Likewise, it is possible that some enzymes having a rate-limiting role in glucose metabolism of *G. duodenalis* and harboring cysteine residues critical for activity modulation (e.g., triosephosphate isomerase) (Reyes-Vivas et al., 2007) are also affected by garlic components. Further assays using recombinant or purified target molecules may be useful to provide more data on the effects of garlic components on *Giardia* trophozoites.

The relation between TAC structure and giardicidal activity showed that the increasing numbers of sulfur atoms are important for this effect and remarkably, points out that the presence of the sulfur-attached oxygen atom in ALC accounts for its maximal efficacy. When comparing the relative giardicidal activity of these seven TACs(ALC>DATS>DADS>AM>DAS>SAC>AMS) with its HOCl-scavenging capacity (SAC>DATS>DAS>ALC>DAS>AM>AMS; Argüello-García et al., 2010) it was observed that garlic's TACs have a wide biological mechanisms suggesting that there is a need to choose the best compound for a defined benefit, either therapeutic (e.g., anticancer and antimicrobial) or antioxidant that will finally induce health-promoting activities upon consumption (Capasso, 2013). As examples, AMS could provide low beneficial effects whereas DATS (sell as Dasuansu in China) could give multiple benefits while ALC could also provide several benefits but with a higher efficacy as antimicrobial agent. Nevertheless, it is still necessary to compare these TACs in other systems to obtain compelling data on the effects of TACs. In addition, the molecular descriptor *E*_*HOMO*_ was useful to predict the antigiardial effect of TACs and interestingly it was a partial contributor for HOCl-scavenging activities of these TACs (Argüello-García et al., 2010). Considering that HOMO are electron orbitals that could act as electron donors and LUMO are electron acceptor orbitals in a given molecule, these data suggest that ALC exhibiting the highest *E*_*HOMO*_ value (−211.71 kcal/mol) is a strong electron donor that could hence act as a nucleophile toward parasite's molecules bearing electron-acceptor groups e.g., thiols (R-SH) at a greater extent as compared with all other TACs used leading to trophozoite death.

There is a growing body of information regarding the efficacy of ALC against other protozoan pathogens. For example, in the human intestinal parasite *Entamoeba histolytica* the reported IC_60_ (500 μM; Ankri et al., 1997) is similar to the IC_50_ value obtained for *G. duodenalis* in this work using the same assay (686 μM) again reflecting the cysteine-rich content and thiol–dependent metabolism of these mitosome-containing parasites (Ali and Nozaki, 2007). In the also diplomonad but aerotolerant, hydrogenosome-containing fish parasite *Spironucleus vortens* ALC has a higher IC_50_ value (>1.12 mM; Millet et al., 2011) that is likely linked to a lower susceptibility of its glutathione-based metabolism (Williams et al., 2014) to this TAC. In contrast, the small intestinal chicken parasite *Eimeria tenella* displays a poor susceptibility to ALC (IC_99_ = 11.1 mM; Alnassan et al., 2015) due at least in part to its diverse metabolism highlighted by several shunt-off pathways like the mannitol cycle (Schmatz, 1997).

As far as the mechanism of cytotoxic action of ALC is concerned, its cytolytic, membrane-damaging effects were evidenced by the higher sensitivity of physiological methods (DCC and TBE) and electron microscopy analyses. In contrast with AGE, the MTT reduction assay displayed the lowest sensitivity of all methods used, suggesting that cellular oxidoreductases are not primary targets of ALC. Although ALC behaved as AGE did against trophozoite's integrity, it had a thiol-disulfide exchange capacity that correlated to and accounted for its highest giardicidal efficacy as compared to all other TACs tested. Therefore, the derivatizing reaction mediated by TACs on giardial proteins exposing thiol-groups to form S-allylmerecpto adducts (R-SH → R-S-S-CH_2_-CH = CH_2_) was proven to be a major mechanism promoting trophozoite's death. However, this general reaction of ALC (Rabinkov et al., 1998) could take place at certain proteins that have important implications for cell viability, proliferation, and differentiation in *G. duodenalis*. Among these are cysteine proteases that intrinsically have the catalytic triad Cys-His-Asn. These constitute a repertoire of around 10 proteins in this parasite, participate in host cell damage and are feasible candidates for new therapeutic strategies in giardiasis (DuBois et al., 2006; Ali and Nozaki, 2007; Ankarklev et al., 2010; Gargantini et al., 2016; Cabrera-Licona et al., 2017; Ortega-Pierres et al., 2018). The zymogram anlaysis presented in Figure 4 indicates that garlic TACs are effectively inhibitors of several cysteine proteases in trophozoite lysates with ALC displaying a remarkably higher inhibitory action than DADS in good agreement with their thiol-modifying activities determined herein and in recent reports in other parasite models (Waag et al., 2010). Of particular interest was the marked inhibition of the proteolytic activity of giardipain-1, a secreted virulence factor of *G. duodenalis* trophozoites, observed with ALC, DADS, and E64 in zymograms, Giardipain-1 has recently been reported to exert pro-apoptotic effects in epithelial cells monolayers including membrane blebbing, external phosphatidylserine exposure, loss of barrier function, caspase 3 activation, and altered localization of tight junction proteins as occludin and claudin-1 (Ortega-Pierres et al., 2018). Moreover, representative TACs as are ALC, DADS and AM were not only able to reduce the cytopathic effects of giardipain-1 but to display a much more marked effect in trophozoites' viability over epithelial cell integrity. These facts suggest that ALC is a promising candidate in further studies at preclinical level in giardiasis.

Other likely targets of ALC and remainder TACs in *Giardia* are the variable surface proteins (VSPs) that are a family with over 230 members having 10–12% of cysteine residues in their sequence that are involved in antigenic variation and other functions, the high-cysteine membrane proteins (HCMp) family with role in epithelium-trophozoite interactions and cell differentiation along with the iron-sulfur cluster containing protein family (e.g., ferredoxins) involved in redox metabolism. Studies in our group on these interactions are now under progress.

At the level of cellular processes, ALC induced apoptotic-like cell death and arrest at G2 phase with thiol but not oxidative stress as shown by flow cytometry studies. When the effects of ABZ, a potent antigiardial agent currently prescribed, it was observed that ALC also promoted apoptosis-like death as many other antigiardial agents from synthetic or plant resources; nevertheless ALC did not cause ROS formation suggesting that TACs are not significantly metabolized by trophozoites unlike most of prescribed drugs as ABZ, MTZ, furazolidone or nitazoxanide (Gardner and Hill, 2001). On the other hand, the putative induction of thiol stress in *G. duodenalis* trophozoites caused by ALC and other TACs by modification of thiol groups to S-allylmercapto adducts (Figure 3) is similar to that reported in *Escherichia coli*; however, in these bacteria ALC induced oxidative stress as assessed by the upregulation of antioxidant enzymes such as OxyR, alkyl hydroperoxide reductase and thioredoxins 1 and 2 (Müller et al., 2016). Interestingly ALC also induced oxidative stress with the same probe used here (H_2_DCFDA) in the mitochondria-containing protozoan parasite *Leishmania infantum* albeit the absence of phosphatidylserine externalization at cell membrane as assessed by annexin V and positive staining with PI indicated a necrotic process of cell death (Corral et al., 2016). All these studies shed light on the multiplicity of microbicidal effects of ALC regardless of its well-recognized mode of action on cellular thiols.

In the experimental gerbil model of giardiasis ALC demonstrated a potential utility at single doses of 70 (5/6 gerbils cured) and 138 mg/kg (6/6 gerbils cured) while AGE was effective using two doses of 770 mg/kg (5/6 gerbils cured). In our experience ALC by intragastric route shows a lethal dose in gerbils of >400 mg/kg and in other rodents this TAC displays oral lethality at 300 mg/kg (mouse) and 980 mg/kg (rat). In spite of this apparently narrow margin of therapeutic security for ALC, in further studies splitting into two or more days the treatment could reduce these doses. Some encouraging examples on this regard have been reported with experimental infections of mice by systemic protozoa as *Babesia microti* where 30 mg/kg ALC orally for 5 days significantly reduced parasitemia and with *Plasmodium berghei* in which 4 daily doses of ALC at either 9 mg/kg (orally) or 8 mg/kg (intravenously) markedly reduced mortality (Coppi et al., 2006; Salama et al., 2014). Concurrent to these purposes is the possibility to improve the bioavailability of ALC or AGE components at intestinal milieu using carriers such as nanoparticles (Pinilla et al., 2017; Soumya et al., 2017).

In summary, the results obtained in this study highlight the cytolytic mechanism involved in the cytotoxic activity of AGE and particularly TACs as ALC that also produce thiol stress, apoptosis-like cell death as a consequence of targeting multiple proteins in *G. duodenalis* including cysteine proteinase activities as shown in this work. The identification and characterization of interactions of garlic components/derivatives with giardial biomolecules deserves future studies and indicates that ALC is a promising antigiardial agent with concomitant benefits for host health.

## Ethics statement

This study was carried out in accordance with the recommendations of Comité Interno para el Cuidado y Uso de los Animales de Laboratorio (CICUAL) Del Cinvestav, México. According with the Official Mexican Rule (Norma Oficial Mexicana) NOM-062-ZOO-1999. The protocol was approved by the Comité Interno para el Cuidado y Uso de los Animales de Laboratorio (CICUAL) Del Cinvestav, México.

## Author contributions

RA-G and MO-P conceived the work, designed the experiments, and analyzed the data. RA-G and NP-H participated in allicin synthesis. NP-H obtained the values of molecular descriptors. MdlV-A performed all techniques of susceptibility assays. IL-R, AM-C, EM-T, and EE-C evaluated extracts and compounds in the gerbil model of giardiasis. RF-L performed the proteolytic activity (zymograms) assyas, the co-culture assays to analyzed representative TACs as possible inhibitors of the cytolytic effect of giardipain-1 on IEC6 cell monolayers and to determine the effect of representative TACs on epithelial IEC6 cell monolayers. RA-G did the docking of with giardipain-1. AG-R performed the electron microscopy (scanning and transmission) studies. RA-G carried out flow cytometry studies. RA-G and MO-P wrote and corrected the manuscript.

### Conflict of interest statement

The authors declare that the research was conducted in the absence of any commercial or financial relationships that could be construed as a potential conflict of interest.

## Supplementary material

Supplementary Figure 1 Determination of the effect of representative TACs on epithelial IEC6 cell monolayers. Confluent IEC6 cells were exposed for 100 min at 37°C to affinity chromatography-purified giardipain-1 alone (control for cell cytotoxic effect) or to concentrations (in mM) of representative TACs causing inhibition of its protease activity in zymograms (center panels) or corresponding to the MLCs in trophozoites (right panels) of representative garlic TACs (ALC, Allicin; DADS, Diallyl disulfide; AM, Allyl mercaptan). Monolayers were analyzed by Nomarski optics. Asterisks denote zones of monolayer destruction and arrows point to cells undergoing apoptotic death as indicated by membrane blebbing, shrinkage and shape degeneration. Bars: 20 μm.

Supplementary Table 1 Values of electronic and molecular transport descriptors calculated for garlic's TACs used in this study.
