# Supplementary figures and images for: Activity of Thioallyl Compounds From Garlic Against Giardia duodenalis Trophozoites and in Experimental Giardiasis
Source: Front Cell Infect Microbiol. 2018 Oct 15;8:353. doi: 10.3389/fcimb.2018.00353 (PMC6196658; doi:10.3389/fcimb.2018.00353)

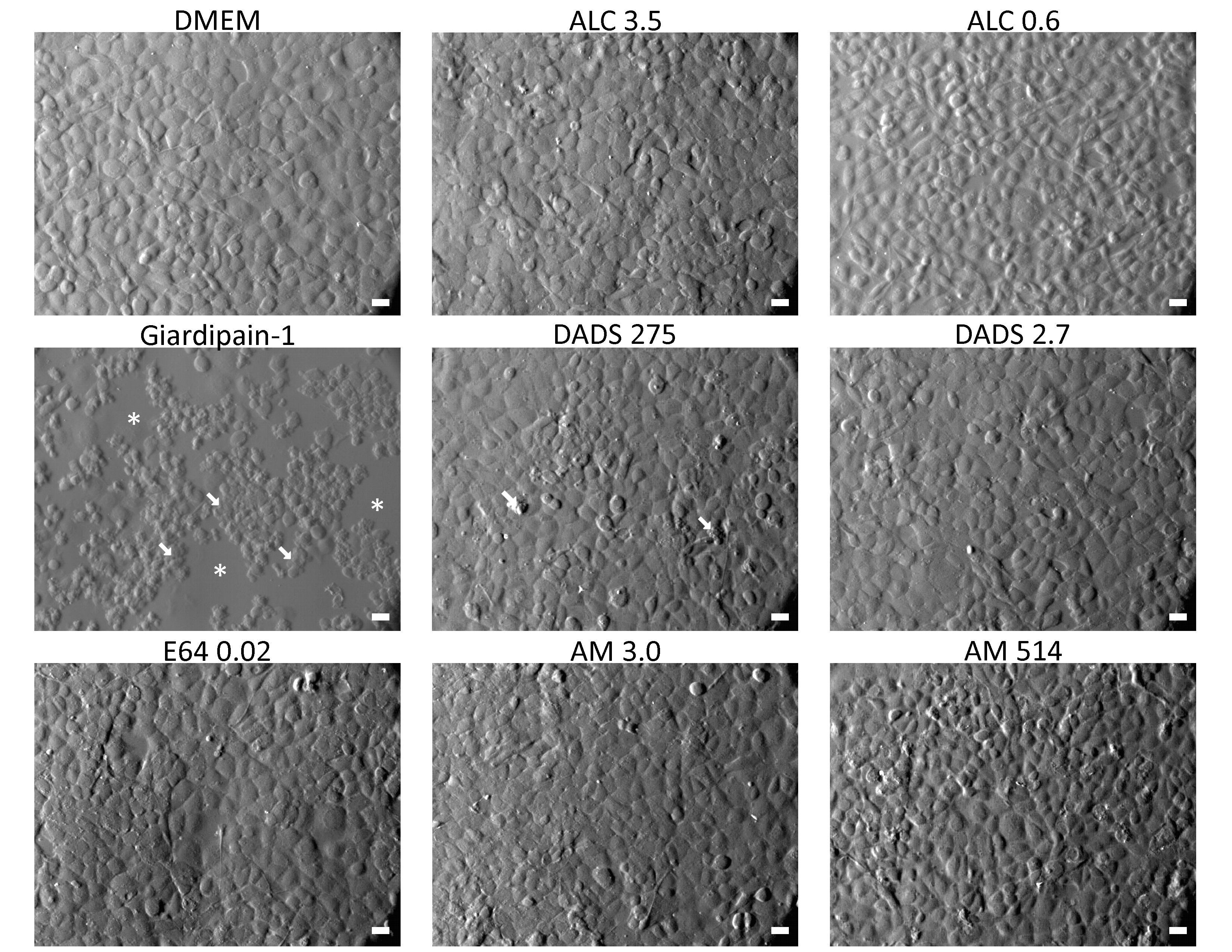

Supplement: Supplementary Figure 1 — Determination of the effect of representative TACs on epithelial IEC6 cell monolayers. Confluent IEC6 cells were exposed for 100 min at 37°C to affinity chromatography-purified giardipain-1 alone (control for cell cytotoxic effect) or to concentrations (in mM) of representative TACs causing inhibition of its protease activity in zymograms (center panels) or corresponding to the MLCs in trophozoites (right panels) of representative garlic TACs (ALC, Allicin; DADS, Diallyl disulfide; AM, Allyl mercaptan). Monolayers were analyzed by Nomarski optics. Asterisks denote zones of monolayer destruction and arrows point to cells undergoing apoptotic death as indicated by membrane blebbing, shrinkage and shape degeneration. Bars: 20 μm. [file Image_1.TIF]
