# Supplementary material for: Activity of Thioallyl Compounds From Garlic Against Giardia duodenalis Trophozoites and in Experimental Giardiasis
Source: Front Cell Infect Microbiol. 2018 Oct 15;8:353. doi: 10.3389/fcimb.2018.00353 (PMC6196658; doi:10.3389/fcimb.2018.00353)
Supplement: Supplementary Table 1 — Values of electronic and molecular transport descriptors calculated for garlic's TACs used in this study. [file Table_1.pdf]

Supplementary Table 1 | Values of electronic and molecular transport descriptors calculated for garlic´s TACs used in this study*.

Thioallyl Compound	E_{HOMO} (kcal/mol)	E_{LUMO} (kcal/mol)	E (kcal/mol)	Dipole (Debye)	TPSA (Å²)	log P_{oct}
Allyl Mercaptan (AM)	-205.72	9.74	13.71	1.78	39.80	1.51
Allyl Methyl Sulfide (AMS)	-195.63	11.54	8.62	1.57	25.30	1.76
Diallyl Sulfide (DAS)	-196.45	8.12	26.38	1.54	25.30	2.75
Diallyl Disulfide (DADS)	-206.96	-36.87	31.57	2.14	50.60	2.91
S-Allyl Cysteine (SAC)	-191.66	12.31	-87.10	2.30	88.62	-2.15
Allicin (ALC)	-212.71	-33.70	-0.78	2.25	61.58	1.52

* Taken from Argüello-García *et al*., (2010)
